# Characterisation and reproducibility of the HumanMethylationEPIC v2.0 BeadChip for DNA methylation profiling

**DOI:** 10.1186/s12864-024-10027-5

**Published:** 2024-03-06

**Authors:** Timothy J. Peters, Braydon Meyer, Lauren Ryan, Joanna Achinger-Kawecka, Jenny Song, Elyssa M. Campbell, Wenjia Qu, Shalima Nair, Phuc Loi-Luu, Phillip Stricker, Elgene Lim, Clare Stirzaker, Susan J. Clark, Ruth Pidsley

**Affiliations:** 1 Garvan Institute of Medical Research, Sydney, NSW 2010 Australia; 2 St Vincent’s Clinical School, UNSW Sydney, Sydney, NSW 2010 Australia; 3 Department of Urology, St. Vincent’s Prostate Cancer Centre, Sydney, NSW 2050 Australia

**Keywords:** DNA methylation, Microarray, EPICv2, WGBS, Validation, Manifest

## Abstract

**Background:**

The Illumina family of Infinium Methylation BeadChip microarrays has been widely used over the last 15 years for genome-wide DNA methylation profiling, including large-scale and population-based studies, due to their ease of use and cost effectiveness. Succeeding the popular HumanMethylationEPIC BeadChip (EPICv1), the recently released Infinium MethylationEPIC v2.0 BeadChip (EPICv2) claims to extend genomic coverage to more than 935,000 CpG sites. Here, we comprehensively characterise the reproducibility, reliability and annotation of the EPICv2 array, based on bioinformatic analysis of both manifest data and new EPICv2 data from diverse biological samples.

**Results:**

We find a high degree of reproducibility with EPICv1, evidenced by comparable sensitivity and precision from empirical cross-platform comparison incorporating whole genome bisulphite sequencing (WGBS), and high correlation between technical sample replicates, including between samples with DNA input levels below the manufacturer’s recommendation. We provide a full assessment of probe content, evaluating genomic distribution and changes from previous array versions. We characterise EPICv2’s new feature of replicated probes and provide recommendations as to the superior probes. In silico analysis of probe sequences demonstrates that probe cross-hybridisation remains a significant problem in EPICv2. By mapping the off-target sites at single nucleotide resolution and comparing with WGBS we show empirical evidence for preferential off-target binding.

**Conclusions:**

Overall, we find EPICv2 a worthy successor to the previous Infinium methylation microarrays, however some technical issues remain. To support optimal EPICv2 data analysis we provide an expanded version of the EPICv2 manifest to aid researchers in understanding probe design, data processing, choosing appropriate probes for analysis and for integration with methylation datasets from previous versions of the Infinium Methylation BeadChip.

**Supplementary Information:**

The online version contains supplementary material available at 10.1186/s12864-024-10027-5.

## Background

DNA methylation, the addition of a methyl (CH_3_) group to a cytosine, most commonly at cytosine-guanine (CpG) sites, is a stable and ubiquitous epigenetic modification in humans. Decades of research have revealed the role of DNA methylation in transcriptional activity and processes including cellular differentiation, genomic imprinting, silencing of repetitive elements and inactivation of the X-chromosome in females [1, 2]. Given these critical roles it is unsurprising that aberrant changes in DNA methylation patterns are associated with cellular dysregulation and disease. This is most evident in cancer where widespread perturbations in DNA methylation are a recognized hallmark of the disease [3]. A substantial body of research also shows the importance of DNA methylation in a wide range of other diseases including autoimmune conditions [4, 5], neurodevelopmental disorders [6] and cardiovascular disease [7]. Moreover, large-scale epigenetic epidemiological studies suggest population-wide associations between environmental exposures, DNA methylation and complex human phenotypes [8]. Tools that can accurately quantify CpG DNA methylation levels across the genome are therefore essential to gain a full understanding of health, disease and treatment opportunities.

A range of technologies are available for quantifying DNA methylation [9, 10]. The most common approaches to distinguish methylated from unmethylated DNA include: sodium bisulphite treatment, which converts unmethylated cytosines to uracils and leaves methylated cytosines unchanged [11] (used in clonal bisulphite sequencing, pyrosequencing, Illumina BeadChips, Whole Genome Bisulphite Sequencing (WGBS)); endonuclease digestion-based methods, which use methylation-sensitive restriction enzymes to fragment double stranded DNA according to its methylation status (e.g. in MRE-seq); affinity enrichment methods that use methylation specific antibodies or proteins to preferentially capture methylated DNA (e.g. in MeDIP-seq, MBDCap-seq); and bisulphite-free base pair conversion methods using oxidation and enzymatic conversion (e.g. in TAPS and EM-seq) [10]. Subsequent sequencing or array hybridisation allows the quantification of the relative amount of methylated and unmethylated cytosines at a particular locus. More recently, nanopore sequencing using a voltage-biased nanopore sensor has enabled direct readout of DNA bases and methylation status [12]. The other major advance has been in single cell methodologies that allow assessment of CpG DNA single molecule methylation in individual cells [13, 14]. Time, cost and human resource constraints inform the investigator’s choice of DNA methylation platform, presenting a need to identify the most reliable methylation measurement strategy particular to their research question.

The Illumina family of Infinium Methylation BeadChips (arrays) have proved popular for their ease of use and cost and have been widely used for genome-wide DNA methylation profiling over the last 15 years. As of December 2023, the Gene Expression Omnibus (GEO) website (www.ncbi.nih.gov/geo) lists the number of series (studies) and samples uploaded for each platform as: HumanMethylation27 (27K, manufactured 2008–2011) – 347 series, 19,056 samples; HumanMethylation450 (450K, manufactured 2011–2016) – 1,707 series, 122,306 samples; and the HumanMethylationEPIC (EPIC, manufactured 2016–2023) – 1,238 series, 79,773. Additionally the European Genome-Phenome Archive (EGA, https://ega-archive.org) and the database of Genotypes and Phenotypes (dbGAP, https://www.ncbi.nlm.nih.gov/gap) repositories each contain 450K and EPIC data from approximately 30,000 samples. To handle this deluge of data different strategies have been developed for data preprocessing and normalization, with R packages such as *minfi* [15], *SeSAMe* [16], *RnBeads* [17] and *wateRmelon* [18] now amongst the most popular. A common approach to analyse processed methylation data is to perform comparisons between disease or treatment groups to identify regions of differential methylation (DMRs), using packages such as *DMRcate* [19] and *BumpHunter* [20]. Identification of DMRs has provided biological insights and paved the way for clinical applications, including biomarker identification and treatment monitoring [21]. The quantity of data generated has also enabled the development of a range of DNA methylation based algorithms to predict phenotypes including age, smoking history, mortality risk, cell type fraction, Body Mass Index and alcohol intake (see *Ori *et al. [22] for a detailed summary and recommendations for their usage). These prediction tools further demonstrate the potential of the methylation arrays for clinical applications.

All versions of the Infinium methylation arrays employ the same bead technology. Oligos with a 23 base address and probe sequence designed to be complementary to specific 50 base regions of bisulphite converted genomic DNA are affixed to beads. After hybridisation of bisulphite converted DNA, single base extension of the probe with a fluorescently labelled dideoxynucleotide triphosphate (ddNTP) allows the assessment of the target cytosine (C) CpG site. Measurement of the fluorescence signal detects the C as thymine (T) if originally unmethylated and converted from C to T by bisulphite treatment and whole genome amplification, or C if methylated and unconverted (see *Pidsley *et al. for a full description of the technology [23]). The amount of DNA methylation at an individual locus is calculated as β = C/(C + T + 100), where C and T are the respective methylated and unmethylated signal with an offset of 100 added to the denominator to avoid infinite values. The resulting methylation beta-value (β) ranges from 0 to 1 (or 0% to 100%).

The original HumanMethylation27 BeadChip (27K array) array featured 25,578 probes primarily targeting CpG sites in promoter regions and known cancer genes. The design was particularly focused on CpG dense regions of the genome called ‘CpG islands’ which are known to be important in gene regulation [24]. The subsequent HumanMethylation450 BeadChip (450K array) increased content to 485,577 probes, targeting 94% of the 27K CpG sites whilst extending to CpG sites in: CpG island shores and shelves; the 5’UTR, 3’UTR and bodies of RefSeq genes; FANTOM4 promoters; the MHC region; and some enhancer regions [25]. The succeeding HumanMethylationEPIC BeadChip (hereafter referred to as EPICv1) almost doubled the probe content to 866,836 probes, which overlapped ~ 90% of the 450K array, plus CpG sites at enhancers identified by the FANTOM5 and ENCODE project [23]. The latest addition, the HumanMethylationEPIC v2.0 BeadChip (hereafter referred to as EPICv2), claims to target over 935,000 CpG sites in biologically significant regions of the human methylome.

The new iterations of the Infinium methylation arrays have not been without technical challenges. The improved coverage of the 450K and EPICv1 array at less CpG dense regions necessitated the introduction of a new type of probe (‘Type II’ probes) which use a different chemistry and produce a different signal intensity distribution that needs to be considered in analysis [18, 26]. A proportion of probes have also been identified as cross-hybridising to multiple regions of the genome or targeting genetically polymorphic sites [23, 27–30]. Annotation files and techniques were developed to take account of these probes which can otherwise lead to incorrect DNA methylation quantification [23, 27–30].

In the current study, we have comprehensively characterised the EPICv2 array and explored its reproducibility for DNA methylation profiling. We provide a full assessment of probe content, including genomic distribution and probe representation compared to previous array versions. We conducted empirical analysis of 40 DNA samples, including replicates, from a variety of sources, including cell lines and patient samples, chosen as representative of the types of samples typically profiled on Infinium arrays. With this data we show high concordance between technical replicates at a range of DNA input levels. Cross-platform analysis of 18 matched DNA samples shows a high degree of reproducibility between EPICv1, EPICv2 and WGBS. We have flagged potentially cross-hybridising probes and their off-target sites, this time at single nucleotide resolution, and provided empirical evidence for the degree of this cross-hybridisation via comparison to the sample-matched WGBS measurements. Finally, we have used our results to construct a new version of the manifest with added information that will aid researchers to better understand the array, choose the best performing probes and integrate EPICv2 data with methylation datasets generated with previous versions of the Infinium Methylation arrays.

## Methods

### Initial characterisations of the Illumina EPICv2 manifest

The Illumina manifest file EPIC-8v2-0_A1.csv was downloaded from Illumina.com 15th Nov 2022 and imported into R 4.3.0 for characterisation. COSMIC census data was downloaded from https://cancer.sanger.ac.uk/census/ 10th March 2023 [31]. The overlap of genomic positions between ‘nv’ probes and COSMIC data was determined using the *GenomicRanges* R package (version 1.52.0) [32]. For the identification of replicate probes within EPICv2, three types of replicate probe were defined using the following criteria: 1) ‘exact-replicate’ – identical probe name and probe sequence, 2) ‘location-replicate’ – identical probe name and different probe sequence, 3) ‘sequence-only-replicate’ – different probe name and identical probe sequence.

### Manifest comparison between EPICv2 and older arrays

‘SeSAMe’ manifest files, ‘Manifest with mapping information’ (hg38) EPICv2, EPIC, HM450 and HM27, were downloaded from https://zwdzwd.github.io/InfiniumAnnotation [16]. This data source was chosen for two reasons. Firstly, unlike the Illumina manifests for 450K and 27K, each manifest file has been re-mapped to the hg38 reference genome which allows for comparison of probe locations between manifests. Secondly, the *SeSAMe* package was, at the time of analysis, the only available software that had integrated the EPICv2 manifest into its preprocessing workflow. To check the agreement between the Illumina EPICv2 manifest and SeSAMe EPICv2 manifest we first matched the two datasets on IlmnID. All probes were common between the two manifests, except for the 824 ‘nv’ probes that were absent from the SeSAMe EPICv2 manifest. Next we compared the Illumina EPICv2 manifest hg38 genomic positions with the SeSAMe EPICv2 manifest hg38 genomic positions. We note that the Illumina EPICv2 manifest gives the coordinate of the single cytosine (‘C’) base in each target CpG site, whereas the SeSAMe manifests give a two nucleotide width coordinate range using the coordinate system in the BED format. To take the different coordinate system into account we used *GenomicRanges* (version 1.52.0) [32] to intersect the mapping information between the EPICv2 manifest files, which revealed 627 probes mapped to different locations (note, the 6,889 probes missing chromosome information in Illumina manifest were removed for this analysis). We have included the SeSAMe genomic coordinates and flagged these probes with discrepant locations in our new version of the EPICv2 manifest. Comparison with the EPICv2 ‘Mask information’ file from https://zwdzwd.github.io/InfiniumAnnotation [16] shows that 99.8% of these discrepant probes are recommended for masking due to low quality of mapping, as well as disproportionately enriched (*n* = 65) for in silico cross-hybridisation via BLAT.

As 99.9% of probe locations agreed between Illumina and SeSAMe EPICv2 manifests we proceeded to use the complete set of SeSAMe manifests for the comparison of array versions. Probes were matched between arrays according to three criteria: 1) probe name, 2) hg38 location and 3) probe sequence. For probe sequence matches both Allele A and Allele B probe sequences were required to match. The matching of probes between different array versions was complicated by probe replicates. Our approach was to treat each EPICv2 replicate probe independently and provide the details of *any* match in the new manifest field ‘EPICv1probeID’. For example, ‘exact-replicate’ probe cg20029347_TC11 and cg20029347_TC12 matches twice to the same probe (cg20029347) in EPICv1. Therefore, this EPICv1 probe name has been duplicated in the new field ‘EPICv1probeID’. ‘Location-replicate’ probes cg09085639_BC11 and cg09085639_BC21 in EPICv2 both match to the same location as EPICv1 probe cg09085639, so again this EPICv1 probe name has been duplicated in the new field ‘EPICv1probeID’. But extra detail in the fields ‘EPICv1locmatch’ and ‘EPICv1seqmatch’ show that only cg09085639_BC21 is a sequence match. Our analysis also revealed a small number of ‘location-replicates’ and ‘sequence-only-replicates’ within the older arrays. We have included additional fields ‘K450locmatch2’ and ‘K27locmatch2’ in our new manifest which give the older platform probe name that overlaps with the replicate probes.

To assess the overlap and differences in probe target locations between EPICv2 and older array versions we compared the unique probe locations between each and visualized using the *ggVennDiagram* (version 1.2.2) package in R [33]. To assess the overlap between probes that were excluded or retained between EPICv1 and EPICv2, with probes previously identified as having technical problems we used the list of EPICv1 masked probes in “EPIC.hg38.mask_sesame.txt” downloaded from https://zwdzwd.github.io/InfiniumAnnotation [16]. In this file, probes in the ‘MASK.general’ column are recommended for masking due to technical reasons including the potential for a SNP to cause switching of channel colour compared to the reference annotation, overlap of the sequence of the probe body with non-unique sequences and the presence of known SNPs within 5 bp of the target site.

### Genic distribution of probe target sites

We determined the genomic distribution of probe locations relative to specific genic features in hg38: known genes, CpG islands, enhancers and super-enhancers. For genic regions we used Gencode data Release 25 (GRCh38.p7), downloaded in February 2017 from https://ftp.sanger.ac.uk/pub/gencode/Gencode_human/release_25/gencode.v25.annotation.gtf.gz. Transcription start site, gene body and intergenic regions were extracted from the gtf file using bedtools. For CpG islands a bed-formatted annotation file of CpG island elements was downloaded from UCSC using the *rtracklayer* Bioconductor package [34]. CpG shores were defined as the regions 2000 bp either side of each CpG island, and all genomic regions > 2000 bp distant were defined as non-CpG. Enhancer elements were downloaded in April 2023 from the FANTOM5 enhancer atlas https://fantom.gsc.riken.jp/5/datafiles/reprocessed/hg38_latest/extra/enhancer/ [35], comprising 63,285 enhancers from 1,829 human tissue samples. Human super-enhancer elements were downloaded in March 2023 from the SEdb 2.0 database http://www.licpathway.net/sedb/download.php [36], comprising 331,146 unique super-enhancer regions derived from 541 human tissue samples. All genic distribution visualisations were created using *ggplot2* (version 3.4.1) [37] or base R.

### Biological resources

LNCaP prostate cancer cells (ATCC, CRL-1740) were cultured as described previously [38]. Normal prostate epithelial cells (PrEC, catalogue no. CC-2555; Cambrex Bio Science) were cultured according to the manufacturer’s instructions in prostate epithelial growth medium (PrEGM, catalogue no. CC-3166; Cambrex Bio Science) as described previously [39]. Genomic DNA for both cell lines was extracted using QIAamp DNA Mini and Blood Mini kit following the manufacturer’s protocol for cultured cells (Qiagen). Breast PDX samples were obtained from experiments approved by the Garvan Institute of Medical Research Animal Ethics Committee (HREC #14/35, #15/25). PDX models were generated and treated with Decitabine and genomic DNA was extracted using the Qiagen QIAamp DNA Mini Kit, as described in *Achinger-Kawecka *et al. *(2024)* [40]. MCF7 and TAMR cells kindly provided by Dr Julia Gee (School of Pharmacy and Pharmaceutical Sciences, Cardiff University), were cultured and DNA extracted using the QIAamp DNA Mini and Blood Mini kit, as described in *Achinger-Kawecka *et al. *(2020)* [41]. TAMR cell lines were treated with Decitabine and genomic DNA was extracted using QIAamp DNA Mini kit, as described in *Achinger-Kawecka *et al. *(2024)* [40].

For the prostate cancer tissue, six patients who had undergone surgery for localised prostate cancer were identified from the Garvan Institute/St Vincent's Prostate Cancer biobank with informed consent and human ethics approval (SVH File Number 12/231). Fresh-frozen tissue was retrieved and matched H&E slides reviewed by a specialist pathologist to identify areas of tumour. DNA was extracted from regions of high tumour content using the Qiagen AllPrep kit (Qiagen).

## Bisulphite conversion and Infinium arrays (EPICv2 and EPICv1)

DNA was treated with sodium bisulphite using the EZ DNA methylation kit (Zymo Research, CA, USA). DNA methylation was quantified using the Illumina Infinium HumanMethylationEPIC BeadChip and HumanMethylationEPIC v2.0 BeadChip (Illumina, CA, USA) run on an Illumina iScan System (Illumina, CA, USA) using the manufacturer’s standard protocol.

## Whole genome bisulphite sequencing

Whole genome bisulphite sequencing libraries were prepared for prostate cancer tissue samples using the CEGX TrueMethyl Whole-Genome kit (v3.1) (Cambridge Epigenetics, UK) with minor improvements as described by *Nair *et al. [42]. Libraries were run on the HiSeq X Ten with one sample per lane. Sequencing reads were mapped to hg38 following the processing steps described by *Pidsley *et al. [43].

## Publicly available genome-wide DNA methylation data

Previously published methylation datasets from our group were used in the cross-platform technical analysis. Data was sourced in house and corresponds to data in GEO repositories (www.ncbi.nih.gov/geo): LNCaP and PrEC WGBS GSE86832 (lifted over from hg19 to hg38), EPICv1 GSE86831; MCF7 and TAMR WGBS GSE118714. Decitabine treated PDX WGBS GSE171074, EPICv2: GSE216989.

## Within-platform analysis

All within-platform analysis was conducted in R. EPICv2 data was processed using the *SeSAMe* package and annotation [16] following the canonical preprocessing pipeline outlined in: https://www.bioconductor.org/packages/release/bioc/vignettes/sesame/inst/doc/sesame.html, including normalisation via *noob*. Detection p-values were extracted using the *pOOBAH* function with a threshold of 0.05. Plots were made using functions in: *ggplot2* (version 3.4.2) [37] for the SNP heatmap; *minfi* (v 1.4.60) [15] for MDS and methylation density plots; *corrplot* (version 0.92) [44] for correlation matrix plots; and base R for scatter plots. EPICv2 bedGraph files were exported and the Integrative Genome Viewer [45] was used for visualization of methylation at prostate epithelial enhancer regions (from the FANTOM5 enhancer atlas, ‘Prostate Epithelial Cells’ (Sample IDs: CNhs10882, CNhs11972, CNhs12014)). The Relative Log Expression analysis was applied to the M-value methylation measurements, following the approach outlined by *Maksimovik and colleagues* [46].We used the *limma* Bioconductor package [47] to identify differentially methylated positions (DMPs) between sample groups with adjusted p-value cut-off of < 0.05 and absolute mean β-value difference > 5%. The *cnSegmentation* and *visualizeSegments* functions in *SeSAMe* were used to perform and visualize copy number segmentation on LNCaP and PrEC EPICv2 data of different DNA input levels.

## Cross-platform analysis

Both EPICv1 and EPICv2 data were processed using the *SeSAMe* package and annotation [16]. *M*-values (logit2 transform of beta) were used from Illumina arrays, and WGBS values were defined as $logit2\left(\frac{C+0.5}{C+T+1}\right)$ where *C* and *T* indicate methylated and unmethylated reads per CpG site, respectively. CpG methylation across platforms was matched on genome coordinate position from SeSAMe annotation for Illumina arrays and GRCh38.p12 position for WGBS. For EPICv2 probes interrogating the same CpG site, the probe appearing first in the Illumina manifest was used. For quality control purposes, probes for which any of the 18 samples in either array returned a detection *P* value > 0.05 were discarded. For both LNCaP and PrEC 500 ng samples, the technical replicate with the fewest probes with detection *P* value > 0.05 was chosen for the analysis. Similarly, any CpG sites from WGBS that had zero coverage in one or more samples were also removed. Probes flagged by SeSAMe as cross-hybridising, however, were retained for the analysis. This resulted in a full row, column and platform complement of 586,916 CpG sites (63% of all CpG probes on the EPICv2 array) on which the cross-platform analysis was performed using the *consensus* Bioconductor package [48], to calculate sensitivity (the platform-wise regression slope from the consensus fit) and precision (the platform-wise residual scatter around the regression line; noting that smaller values are superior) for each site.

## Mapping of cross-hybridising sites

We identified putative cross-hybridising sites from EPICv2 probes in a manner similar to *Pidsley *et al. [23]. Probe sequences were mapped using BLAT [49] to four versions of human genome reference GRCh38.p12: forward and reverse, bisulphite converted and non-converted. Alignments with 47 or more nucleotide matches (resulting in a BLAT score ≥ 44) were reported as cross-hybridising. In this analysis, we have changed the definition of an in silico cross-hybridisation event to include indels as well as substitutions, whereas previously we only allowed the latter [23]. We note that this change resulted in only 3.35% extra BLAT hits than if we would have replicated our method exactly for *Pidsley *et al. [23], and applying the new method including hits with indels to EPICv1 probes would have resulted in an additional 2.58% hits for EPICv1 probes.

Probes that had no chromosomal coordinate (*n* = 6,889, reported in the Illumina manifest as CHR = chr0 and MAPINFO = 0), no homology above the aforementioned threshold to any of the reference genomes (*n* = 24), or no homology to the stated MAPINFO site (*n* = 18) were excluded from this analysis. For each BLAT hit, we transposed the relationship between the on-target alignment and its reported CHR/MAPINFO cytosine coordinate to the off-targets from the same probe sequence, and reported the coordinate of the corresponding nucleotide. For a subset of cross-hybridising probes (*n* = 17,928), we inferred the level of cross-reactivity by calculating the root mean squared error (RMSE) between the EPIC measurements and the matching WGBS measurements for both on-target and off-target CpGs using the same data as the cross-platform analysis. This calculation required an EPICv2 probe to have at least one CpG off-target, and at least 12 out of 18 matched methylation values retained across both array and WGBS (dropouts incurred by detection *P* > 0.05 on EPIC, or zero coverage on WGBS). For probes whose RMSE with at least one off-target CpG was lower than the target, the off-target with the lowest RMSE was reported as a suggested alternative.

## Competitive evaluation of replicates

Replicate probes were grouped into replicate probe sets by name, sequence or chromosomal location, as described above. Where there was a target site match on EPICv1 and at least 12 out of 18 matched methylation values across all three platforms (same criteria as for the cross-hybridisation analysis), we performed a set of cross-platform analyses, again using *consensus* [48] for 1) each replicate probe and 2) each replicate probe set using their sample means. If the replicate probe had both superior sensitivity *and* precision compared to the other replicate probes, as well as the probe set mean, it was denoted as a “superior probe”. Conversely, if the replicate probe had inferior sensitivity and precision to all other probes in the set, as well as the probe set mean, it was denoted as an “inferior probe”. If different probes in a probe set were superior in terms of sensitivity or precision only, they were individually labelled as “best sensitivity” or “best precision”. It is important to note that the sensitivity (slope of the row-linear fit) of the probe set mean is, by definition, intermediate of the most extreme sensitivities of the individual probes in that group. However, this constraint does not hold for precision (scatter around the row-linear fit), and hence those probes which do not have superior sensitivity in the probe set, but where the probe set mean nonetheless confers the best precision, are labelled “best precision by group mean”. Additionally, the probe with the best sensitivity in such a group is labelled “best sensitivity” regardless of whether the probe set mean is the best in terms of precision. If no matched EPICv1 data was available, a probe was denoted as “superior by WGBS” or “inferior by WGBS” by whether their RMSE with WGBS was the smallest of the probe set or not, respectively. Probes were relabelled “Superior group mean (by WGBS)” if the RMSE of the sample means outperformed all other individual probes in that probe set. Remaining replicate groups were flagged as having insufficient evidence for evaluation.

## Results

### Categorisation of EPICv2 probe types

EPICv2 has increased the number of probes to 937,690 from EPICv1 (866,836), 450K (485,577) and 27K (25,578). To enable an initial characterization of the new probe content featured on EPICv2 we first explored the data provided in the Illumina manifest file. There are four key probe types described in the manifest, distinguished by a ‘locus target identifier’, which is the first two letters of the probe name: ‘cg’, ‘ch’, ‘rs’ and ‘nv’ (Additional file 1: Table S1). ‘cg’ probes (*n* = 933,252) measure cytosine methylation levels at CpG sites and represent the majority of probes on the array. ‘ch’ probes (*n* = 2,914) measure cytosine methylation levels at CpH sites (CpA, CpT or CpC). ‘ch’ probes were first included in the 450K array in recognition of the importance of non-CpG methylation in embryonic stem cells [25]. ‘rs’ probes (*n* = 65, of which 62 are unique) measure genotype at the corresponding Single Nucleotide Polymorphism Database (dbSNP) rsID. These are common human SNPs that can be used for quality control purposes [24]. ‘nv’ probes (*n* = 824) measure genotype at 474 unique ‘nucleotide variant’ or ‘new variant’ loci. These sites are Single Nucleotide Variants (SNVs) that do not have an rsID and were not featured in EPICv1 but are newly included to EPICv2 to target common cancer driver mutations [50]. We confirmed that 472/474 of the ‘nv’ targeted loci overlap with cancer driver genes in the COSMIC “Cancer Gene Census” list [31] (for annotation of ‘nv’ probes with COSMIC data see Additional file 1: Table S2). All probes can be further categorised by their ‘Infinium Design Type’, with the majority using the Type II design (Additional file 1: Table S1). An additional 635 control probes indicate the success of different steps of the experiment and can be used for troubleshooting such as identifying problems with bisulfite conversion, probe hybridisation or staining (Additional file 1: Table S3) (see manufacturer’s guide for full description of control probes: [51]).

### Explanation of new EPICv2 probe naming convention

**Fig. 1:**
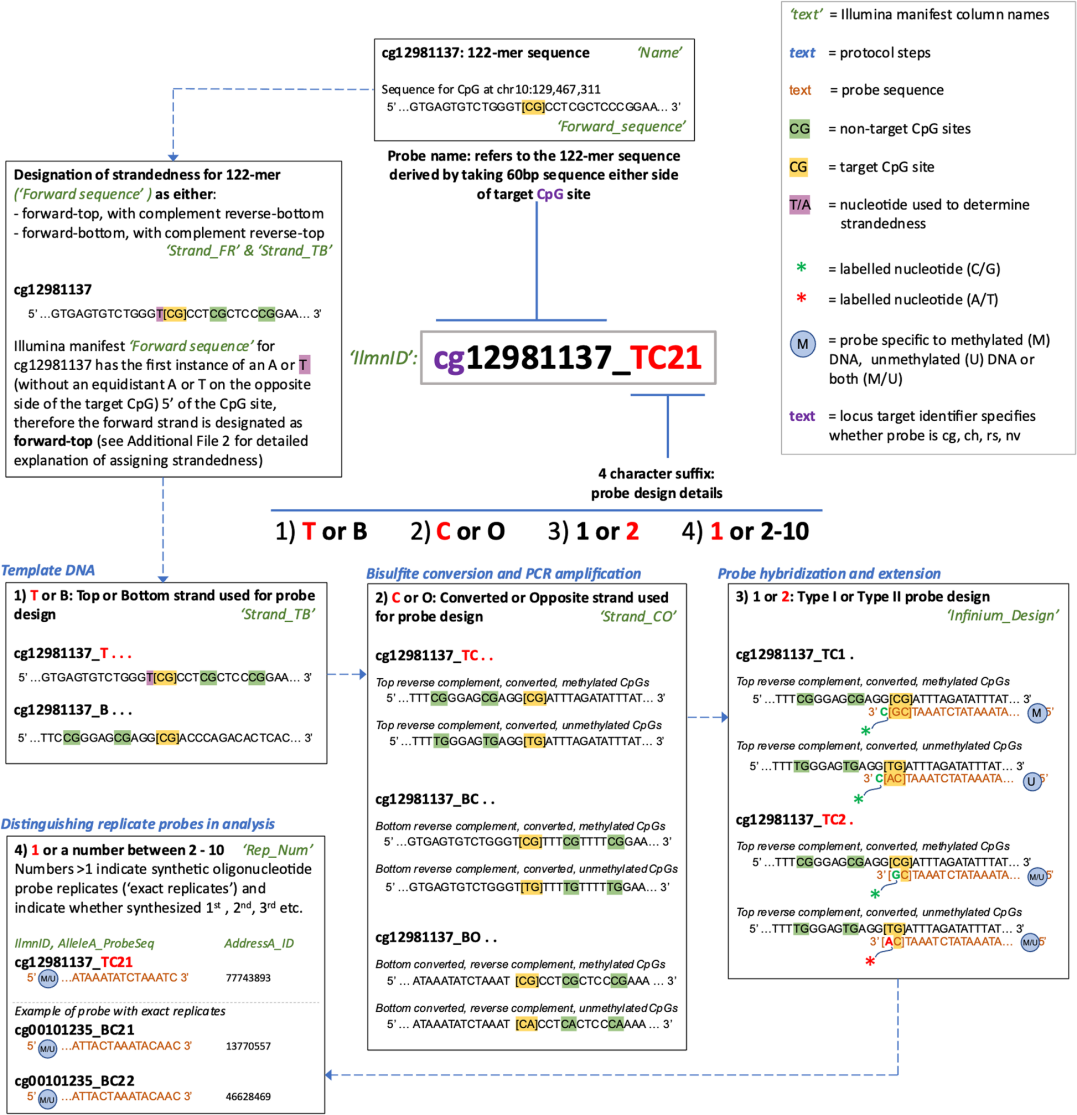
Schematic of EPICv2 probe design demonstrating relationship between IlmnID and probe sequence

We next examined the meaning of the new naming convention of the EPICv2 probes, beginning with information from an Illumina technical document: [52]. In each previous version of the Infinium methylation microarrays each probe had a unique design, targeted a unique site in the genome and had a unique probe ‘Name’: a two letter ‘locus target identifier’ followed by an eight-digit number (e.g. cg09617579). A new feature of EPICv2 is that some sites in the genome are targeted by multiple probes, some of which have identical probe design (replicate probes). A new probe naming convention on EPICv2 allows each probe to still have a unique probe name, even replicate probes targeting the same locus. Briefly, in the Illumina EPICv2 manifest the ‘IlmnID’ comprises the ‘Name’ and a new four-character suffix (e.g. ‘cg09617579_BC12’). Each of the four characters in the suffix encodes additional information about the probe: 1) whether designed to the top (T) or bottom (B) strand, 2) whether designed to the bisulphite converted strand (C) or opposite strand (O), 3) the Infinium probe design Type I (1) or Type II (2), and 4) the number of times the exact same probe has been synthesized for representation on the array (equivalent to the ‘Rep_Num’ field in the Illumina manifest). Figure 1 and Additional File 2 provide a step-by-step description of our interpretation of the relationship between the IlmnID and probe design. It is worth noting that in the first letter of the IlmnID suffix the top (T) or bottom (B) strand do not refer to the plus and minus strand in the reference genome database, as may be expected, but instead are assigned by Illumina for each sequence fragment individually based on a sequence walking method. To demonstrate the relationship between the IlmnID suffix information and the ‘Forward sequence’ provided in the Illumina manifest, we have created a bioinformatic pipeline that is able to recompute the probe sequence correctly for 98.43% of the probes on the array (see Additional File 3; examples and instructions in Additional File 2 and the 1.57% discrepant probe sequences in Additional File 1: Table S4). We also note that the ‘Next_Base’ and colour channel fields in the Illumina manifest provides information for Type I probes about the nucleotide immediately following the target CpG site. For the majority of probes this is the probe extension site, but for Type I probes designed on the opposite (‘O’) strand the extension site is instead the C of the target CpG site. We therefore recommend using the SeSAMe manifest “Color_Channel” and “Col” information (downloaded from https://zwdzwd.github.io/InfiniumAnnotation) [16] when processing raw signal intensities for data analysis, as this data is derived from the probe extension site (see Additional File 2 for examples).

### Replicate probes on EPICv2

As replicate probes are a new feature of the EPICv2 array, bioinformatic approaches developed for previous versions of the Infinium methylation array may require updates to accommodate them. For example, methods for identifying DMRs, such as R package *DMRcate* [19], or for integrating methylation data from different Infinium methylation array versions, such as R package *meffil* [53], would need to take account of multiple probes targeting the same location. To inform such bioinformatic analysis we explored and described the replicate probes on EPICv2. According to the EPICv2 Illumina manifest ‘Rep_Num’ field there are 5,141 probes, that each have between 2–10 replicates (Additional File 1: Table S5a). These probes are exact replicates, that is sets of probes with the same ‘Name’ (cgXXXXXXX) and the same probe sequence (Additional File 1: Table S6). We also identified additional replicate probe sets defined by 1) a shared target location but differing probe sequence or 2) a shared probe sequence but differing ‘Name’, probes of this replicate type are a subset of 6,889 probes in the manifest that are lacking chromosome mapping information for unknown reasons. For a full description and list of these different replicate probe types and how they are represented within the new probe naming system see Additional File 1: Table S5-8 and Additional File 2). Importantly, to summarise the insights from our replicate probe analyses we have created a new version of the manifest (Additional File 4) with additional fields that allow identification of the different types of replicate probes and replicate number (‘namerep’, ‘seqrep_IlmnIDs’, ‘seqrep_RepNum’, ‘posrep’, ‘posrep_IlmnIDs’, ‘posrep_RepNum’) (see Additional File 5 for description of new field headers).

### Comparison of probe content with previous Infinium methylation array versions

To enable cross-platform integration and comparison, and evaluation of changes in the genomic distribution of probes, we next compared the probe content of the EPICv2 array with the content of previous versions of the Infinium methylation arrays. The Illumina EPICv2 manifest contains fields indicating, for each probe, whether it targets a loci also targeted by previous arrays: a) Methyl27_Loci, b) Methyl450_Loci, c) EPICv1_Loci. These fields indicate overlap between EPICv2 and a) 24,490 27K loci, b) 395,936 450K loci and c) 727,222 EPICv1 loci. Notably these fields also contain the name of corresponding probes in the previous arrays, which reveals that some probe names have changed between versions, making a simple overlap of probe names insufficient for comparing array content. Furthermore, we found these fields to be incomplete when matching the EPICv2 manifest with previous manifests by 1) probe name, 2) hg38 location and 3) probe sequence (Additional File 1: Table S9 & 10). As a helpful resource we have added the results of the probe matching between array versions, as additional fields in our new updated version of the manifest (see Additional File 4 alongside Additional File 5 for description of new field headers).

**Fig. 2:**
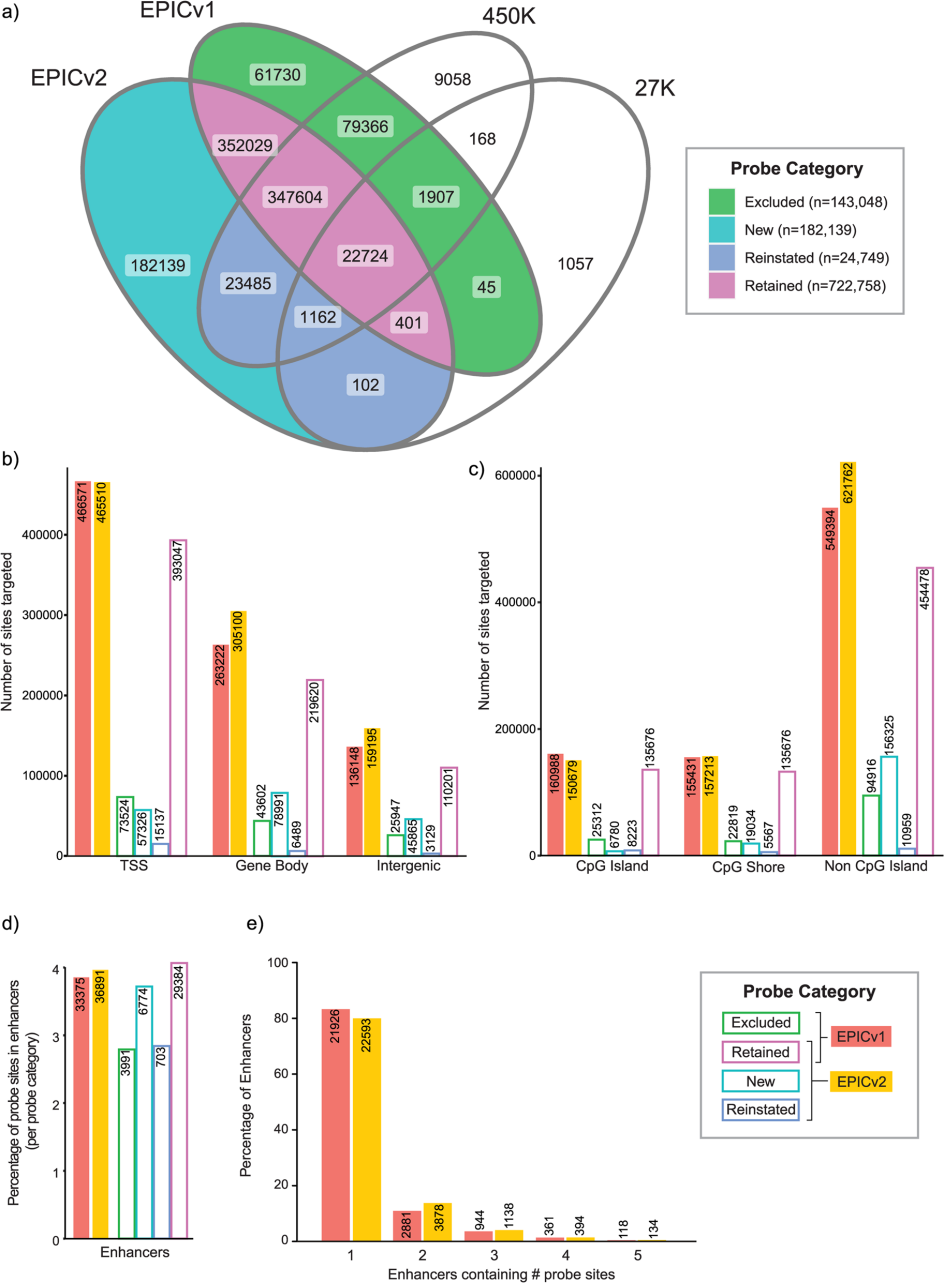
Distribution of probes on EPICv2 compared to older platforms. **a**) Venn diagram showing overlap in sites targeted by different versions of the Infinium methylation microarray (excludes nv and rs loci). Overlap used to define probe categories: Excluded, Retained, New and Reinstated. Number of sites targeted by each probe category relative to **b**) genic and **c**) CpG island context. **d**) Percentage (bars) and number (labels) of probe sites per probe category overlapping FANTOM5 enhancers. **e**) Percentage (bars) and number (labels) of FANTOM5 enhancers containing specified number of probe sites

We next used the results of the cross-platform probe matching to quantify the overlap in content between the four array versions. First, we considered the SNP and CpH loci and observe that the majority are common between the 450K, EPICv1 and EPICv2 (Additional File 6: Figure S1). Next, matching on the unique locations targeted by each array (with ‘rs’ and ‘nv’ probes removed) we show that the majority of locations targeted by EPICv2 were also targeted by previous arrays (747,507, 80.4% of EPICv2 locations), whilst 182,139 (19.6% of EPICv2 locations) are uniquely targeted by the EPICv2, termed ‘new’ (Fig. 2a). Of those locations targeted by previous arrays 722,758 were on EPICv1, termed ‘retained’, whereas 24,749 were on 450K or/and 27K but not EPICv1, termed ‘reinstated’. We also observe that probes targeting 143,048 locations, were removed between EPICv1 and EPICv2, termed ‘excluded’. Illumina’s Product Information Sheet [52] describes that probes with known technical problems including cross-hybridisation [29] were removed between EPICv2 and EPICv1. To verify this we compared the ‘excluded’ probes to the masking files from *Zhou *et al. [29]. We found 72.8% of the ‘excluded’ probes overlapped with probes recommended for masking. In contrast only 0.2% of the probes ‘retained’ between EPICv1 and EPICv2 were found in the mask recommendation list.

## Genomic distribution of reinstated, retained and new EPICv2 probes

### Genic distribution

The EPICv2 probes are distributed throughout the genome (see Additional File 7 and Additional File 1: Table S11). The majority (50.1%) of sites targeted by EPICv2 are located in transcription start sites (TSS), followed by 32.8% in gene bodies and 17.1% in intergenic regions (Additional File 1: Table S12). Compared to EPICv1 the percentage and absolute number of sites in TSSs is reduced (EPICv2 50.1% to EPICv1 53.9%, a reduction of 1,061 sites), as well as the number of unique TSSs covered (EPICv2 58,322 to EPICv1 58,481), whereas sites in gene bodies and intergenic regions are increased (gene body EPICv2 32.8% to EPICv1 30.4%, increase of 41,878 sites, and intergenic EPICv2 17.1% to EPICv1 15.7%, increase of 23,047 sites) (Additional File 1: Table S12, Fig. 2b and Additional File 6: Figure S2a). This change in genic distribution is in accordance with the relatively high proportion of ‘new’ sites targeted in intergenic (‘new’ 25.2% vs ‘retained’ 15.3%) and gene body (‘new’ 43.3% vs ‘retained’ 30.4%) regions, and a much lower relative proportion at TSSs (‘new’ 31.5% vs ‘retained’ 54.4%). We also note that, a high 61.2% of ‘reinstated’ probe sites are in TSSs, which reflects the original content of the 27K and 450K arrays that were highly biased towards promoter CpG island regions. However, the 15,137 ‘reinstated’ probe sites at TSSs do not increase overall TSS coverage in EPICv2, as 73,524 TSS sites were also excluded between EPICv2 and EPICv1.

### CpG island distribution

The majority (66.9%) of sites targeted by EPICv2 are located outside of CpG islands, with 16.2% located in CpG islands and an equivalent 16.9% in CpG shores, defined as the region 2000 bp either side of a CpG island (Additional File 6: Figure S2b and Additional File 1: Table S12). Compared to EPICv1 there has been a relative increase in the proportion of sites targeted in non-CpG island regions (EPICv2 66.9% vs EPICv1 63.5%), with 85.8% of the sites targeted by ‘new’ probes located in non-CpG islands. The proportion and number of sites targeted in CpG islands has decreased from EPICv1 160,988 (18.6%) to EPICv2 150,679 (16.2%) (Fig. 2c). Furthermore, the number of unique CpG islands covered by EPICv2 (*n* = 25,837) has also decreased compared to EPICv1 (*n* = 26,424) (Additional File 1: Table S12). In EPICv2 a larger proportion of the covered CpG islands are assayed at ≤ 2 sites compared to EPICv1 (EPICv2 21.5% vs EPICv1 17.0% of covered CpG islands assayed at ≤ 2 sites) (Additional File 6: Figure S3). In summary, the number of CpG islands covered and the density of coverage within CpG islands has decreased in EPICv2 vs EPICv1. This is inconsistent with Illumina’s claim to have increased coverage of CpG islands [52]. However, the absolute number of sites targeted in CpG *shores* has increased in EPICv2, despite a reduction in the proportion of sites in shores assayed within each array (EPICv2 157,213 (16.9%) vs EPICv1 155,431 (18.0%)) (Additional File 1: Table S12 and Fig. 2c).

### Regulatory element distribution

To next assess if the new content in EPICv2 targets more enhancers and super-enhancers, as stated by Illumina [52], we compared the targeted sites to the location of enhancers in the FANTOM5 enhancer database. Results showed an increase in the number and proportion of EPICv2 sites (36,861, 4.0%) overlapping enhancer regions compared to EPICv1 (33,375, 3.9%), due to an additional 6,774 ‘new’ and 703 ‘reinstated’ sites in enhancer regions compensating for the 3,991 ‘excluded’ from EPICv1 to EPICv2 (Fig. 2d, Additional File 1: Table S12). This resulted in an increase in both the number of enhancers targeted and the density of coverage within enhancers (EPICv2 28,247 enhancers covered, of which 20.0% contain > 1 probe site, vs EPICv1 26,333 enhancers covered, of which 16.7% contain > 1 probe site) (Fig. 2e). Although, as with EPICv1, the majority of EPICv2 covered enhancers (22,593, 80.0%) are targeted by just 1 probe.

Next, we compared array sites to the location of super-enhancers in the SEdb2 (Additional File 1: Table S12). This showed a modest increase in the number and percentage of super-enhancers covered in EPICv2 compared to EPICv1 (EPICv2 480,074 CpG sites within 300,266 super-enhancers, an increase from EPICv1 445,643 CpG sites within 298,228 super-enhancers). A substantial 51.6% of EPICv2 sites are located in super-enhancers. This likely reflects the fact that super-enhancers are extremely large, so the chances of an array CpG probe site lying within an individual super-enhancer is high. However, we do find some evidence that the new EPICv2 content was designed to target functional regions within super-enhancers as when we analysed the super-enhancer probes by genomic/enhancer region we found more super-enhancer probes at TSSs in EPICv2 (*n* = 242,749) compared to EPICv1 (*n* = 241,724). This is in contrast to the analysis of all probes at TSS which showed a greater number in EPICv1 (*n* = 466,571) than EPICv2 (*n* = 465,510) (Additional File 1: Table S12).

### Performance and reproducibility of the EPICv2 array—within platform analysis

To further evaluate and benchmark the performance of the EPICv2 array we profiled DNA isolated from different sample types with different expected methylation states, including commercially available and well-characterised cell lines: prostate cancer cell line (LNCaP), primary cell cultures of prostate epithelial cells (PrEC), fresh-frozen human prostate tumour samples (PrCa), breast cancer cell lines (MCF7 and TAMR), and human breast cancer patient derived xenografts (PDX). Some of the TAMR and PDX samples had been treated with the hypomethylating drug decitabine (see Additional File 1: Table S13 for details). On EPICv2 the majority of samples passed initial quality control checks, with > 90% CpG sites with pOOBAH detection *p*-value > 0.05 (Additional File 1: Table S14, Additional File 6: Figure S4a). Samples which failed quality control were those with less than the recommended DNA input level (included for technical assessment) and two decitabine treated cell lines, which may be due to decitabine induced DNA damage which would lower sample quality [54]. We also demonstrate the utility of the 65 control SNP probes on the array by confirming that samples were matched as expected, including those that failed detection p-value quality control (Additional File 6: Figure S4b).

**Fig. 3:**
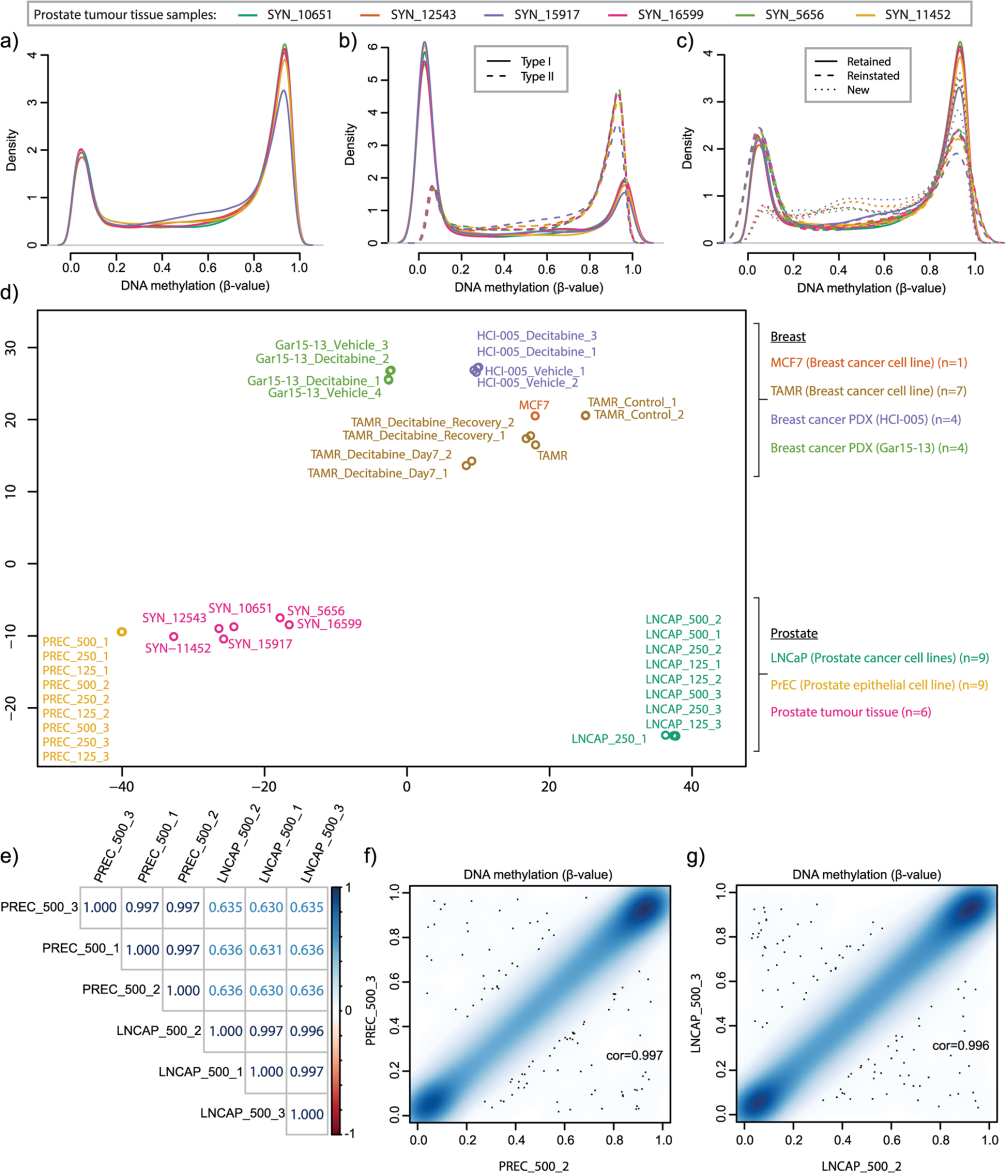
Characterisation and reproducibility of methylation values of samples profiled on the EPICv2 array. Density plots of the methylation β-value of prostate tumour tissue samples for **a**) all probes, and probes separated by **b**) Infinium Design type (Type I or II) and **c**) probe category Retained, Reinstated or New. **d**) Multidimensional scaling plot of all samples across the 10,000 most variable positions. **e**) Correlation matrix of LNCaP (*n* = 3) and PrEC (*n* = 3) technical replicates. Correlation between pairs of **f**) PrEC and **g**) LNCaP technical replicates (with Pearson correlation coefficient and p-value)

DNA methylation β-value density plots showed that, where expected, samples had a bimodal distribution, with the two peaks indicating the unmethylated (0) and fully methylated (1) states typically found in mammalian DNA methylation data (Fig. 3a and Additional File 6: Figure S5). In previous versions of the methylation microarray, differences in methylation distribution were noted between Type I and Type II probes. We plotted the β-values for each probe type separately and observed the same trend in EPICv2 data (epigenetic drug treated samples removed), with the two Type II peaks shifted towards the centre relative to Type I (Fig. 3b and Additional File 6: Figure S6a-d). Next, we performed a comparison of the methylation distribution between probes i) ‘retained’ from the EPICv1, ii) ‘reinstated’ from 27K or 450K array and iii) unique to EPICv2, ‘new’ (Fig. 3c and Additional File 6: Figure S6e-f & 7). Plots show that the methylation distribution of ‘retained’ and ‘reinstated’ probes are largely the same bimodal distribution, likely reflecting the bias of the older array versions to CpG islands and genic regions. New probes (both Type I and II) show the emergence of a third peak at intermediate methylation values, consistent with the design of EPICv2 including more probes targeting enhancer regions, which are known to show intermediate methylation values [55].

We next used the β-value density plots of all probes to confirm that EPICv2 was able to capture the expected relative differences in methylation distributions between cell types, including global hypomethylation in cancer cells [56] and gross hypomethylation in decitabine treated samples [40] (Additional File 6: Figure S5). Comparing all 40 samples together, an MDS plot shows clustering by sample type (Fig. 3d). Samples also cluster by tissue of origin: breast cancer cell lines (TAMR and MCF7) cluster together with breast PDX samples at the top of the plot, whereas prostate cell lines and prostate tumour tissue cluster towards the bottom. This is consistent with the known association between methylation and cell and tissue identity. Together the β-value density and MDS plot show that at the level of global methylation the EPICv2 data is informative, showing the expected clustering and genome-wide differences between samples.

To evaluate EPICv2 technical accuracy we ran a series of technical replicates including LNCaP and PrEC samples in triplicate, distributed across three separate EPICv2 arrays. Initial visualisation showed tight clustering of technical replicates in MDS plots (Fig. 3d) and near identical β-value density plots (Additional File 6: Figure S5b-c). Pairwise correlations showed high correlation between technical replicates (*r* > 0.996, *p*-value < 2 × 10–16) (Fig. 3e-g), indicating that DNA methylation data generated on EPICv2 is highly reproducible across different EPICv2 arrays.

### DNA input analysis

**Fig. 4:**
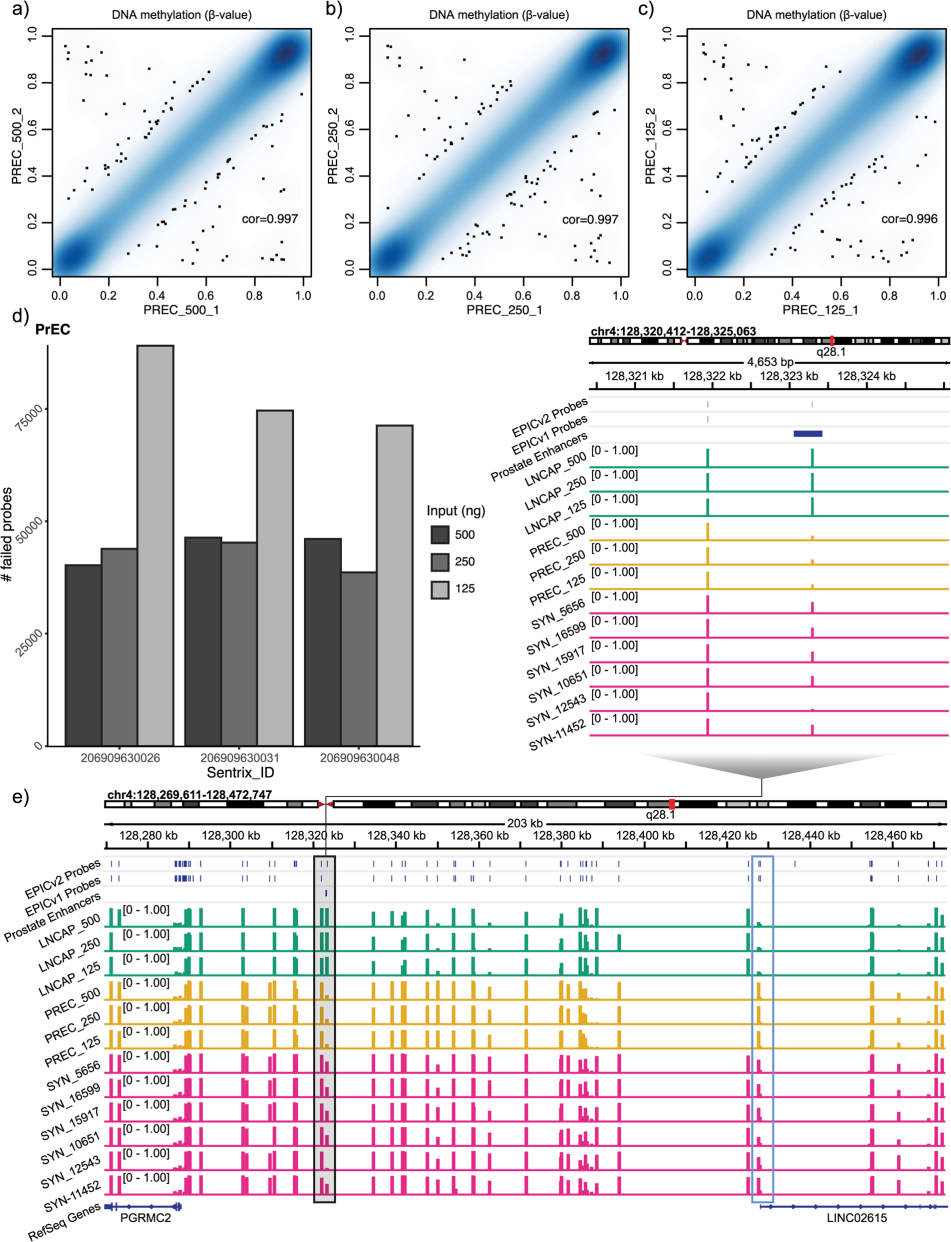
Assessment of reproducibility of methylation values at lower input levels than recommended by the manufacturer. Correlation plots of the methylation β-value of PrEC technical replicates with varying DNA input levels at **a**) 500 ng, **b**) 250 ng and **c**) 125 ng. **d**) Number of probes with detection *p*-value > 0.05 in PrEC technical replicates with varying DNA input levels. **e**) DNA methylation of prostate cancer tissue and cell line samples (of varying DNA input levels) at new EPICv2 sites overlapping newly targeted FANTOM5 prostate specific enhancer (black box) and nearby gene promoter showing inverse methylation between sample types (blue box)

Illumina recommends a minimum of 500 ng per sample for successful methylation profiling with EPICv2. However, clinical samples are often limited, and so for clinical applications it is useful to test the quality of data attainable for lower input levels. We ran additional technical replicates of LNCaP and PrEC DNA diluted to 250 ng and 125 ng (Fig. 4a-c). Quality control using the detection p-value showed that samples had similar quality levels between 500 and 250 ng, with poorer quality data at 125 ng, evidenced by a marked increase in the number of probes per sample failing detection p-value threshold (Fig. 4d and Additional File 6: Figure S4a & 8a). The exception was one LNCaP replicate at 250 ng which showed the highest number of failed probes.

To assess the data quality at different DNA input levels we applied the Relative Log Expression (RLE) method on the M-value methylation measurements, following the approach outlined in *Maksimovik and colleagues* [46]. Relative Log Methylation (RLM) plots show the deviation from the median methylation M-value for each sample. When applied to DNA from LNCaP and PrEC cells separately we observed a slightly larger deviation from the median methylation M-value in the 125 ng samples compared to the 250 ng and 500 ng samples (Additional File 6: Figure S8b-c), suggesting a reduction in data quality at 125 ng.

Pairwise correlations of technical replicates showed high correlations (*r* > 0.995), with only a slight improvement at higher DNA concentrations between the minimum correlation between 125 ng samples of PreC: *r* = 0.9961 and LNCaP: *r* = 0.9956), compared to a minimum correlation between 500 ng samples of PreC: *r* = 0.9970 and LNCaP: *r* = 0.9965 (Fig. 4a-c, Additional File 6: Figure S8d & 9). The similarity between replicates at 125 ng suggests that the data could still be usable for some types of DNA methylation analysis, despite many of the 125 ng samples failing formal quality control checks and showing reduced quality in the RLM analysis.

To test the usability of low input data, we conducted an analysis to compare the number of differentially methylated probes (DMPs) between LNCaP and PrEC DNA at different input levels. Given the failed LNCaP replicate at 250 ng we restricted the comparison to the 500 ng and 125 ng samples. We performed genome-wide analysis of differential methylation using *limma* [47] with Sentrix ID and cell type (LNCaP or PrEC) as variables. As expected, we found a higher number of DMPs (adjusted *p*-value < 0.05 and absolute mean β-value difference > 5%) between the 500 ng LNCaP vs PrEC (*n* = 464,220) than the 125 ng LNCaP vs PrEC (*n* = 425,892), suggesting a reduction in data quality at 125 ng. However, 409,576 DMPs overlapped between the two input analyses indicating that the data from 125 ng samples can be informative.

As further proof of concept, we examined the methylation status of our prostate cell technical replicates, as well as our prostate tumour tissue samples, at normal prostate epithelial cell enhancer regions (*n* = 3,145) from the FANTOM5 collection. We found that 2,144/3,145 prostate enhancer regions were covered by EPICv2, of which 266 were not previously covered by EPICv1. Visualisation of the data at newly targeted enhancer regions shows that there is strong agreement between technical replicates across DNA input levels (Fig. 4e and Additional File 6: Figure S10). For example, at a newly targeted prostate enhancer region between the *PGRMC2* and *LINC02615* genes we see consistent hypermethylation in LNCaP DNA across all DNA input levels compared to consistent hypomethylation PrEC across all DNA input levels, whilst FFPE prostate tumour tissue samples exhibit intermediate levels of methylation (Fig. 4e). Visualisation of methylation at the nearby *LINC02615* gene promoter shows the inverse methylation pattern suggesting a potential mechanistic link between these two regions. This demonstrates the utility of EPICv2 for examining methylation change in clinical tissue and at a range of DNA input levels, at a previously untargeted genomic region. In another proof of concept we used the LNCaP DNA (with PrEC DNA as a reference) to call CNVs using *cnSegmentation* function in the ‘SeSAMe’ package [16] as previously performed on EPICv2 by *Kaur *et al. [57]. This showed the expected LNCaP deletions at chromosome 2p and 13q [58], crucially, across all input levels (Additional File 6: Figure S11).

### Reproducibility of EPICv2 array: cross-platform analysis

**Fig. 5:**
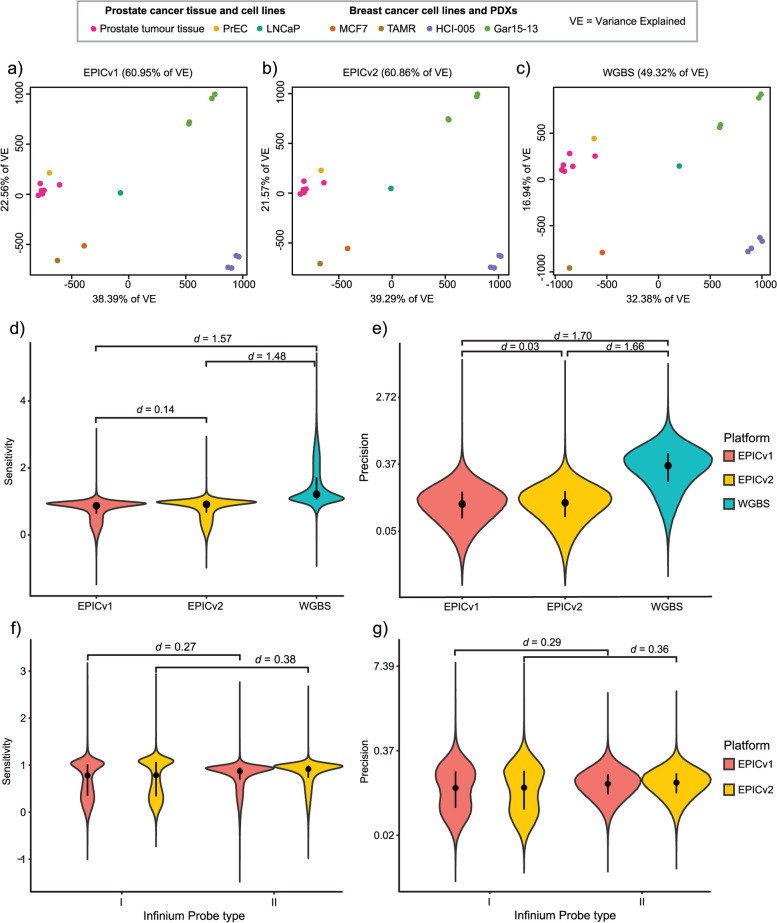
Cross-platform reproducibility of EPICv2. Principal component analysis (PCA) of the matched data (586,916 CpG sites) used in the cross-platform analysis, across **a**) EPICv1, **b**) EPICv2 and **c**) WGBS. Distributions of **d**) sensitivity to methylation change and **e**) precision of methylation measurement for the 586,916 CpG site summaries obtained via the row-linear method. **f**) Sensitivity and **g**) precision of EPICv1 and EPICv2 probes, split by Infinium probe type

Next, we sought to evaluate the performance of EPICv2 relative to other platforms, choosing to use a consensus modelling approach to avoid biasing our comparisons to a particular technology (for more details on the method see *Peters *et al. [48]). This approach is adapted from the American Society for Testing and Materials Standard E691, a method designed to quantify interlaboratory technical variation, although in this instance the variation is inter-platform. The principal outputs of this application are platform-wise summaries of sensitivity and precision relative to the consensus mean. For this analysis we used methylation measurements from 18 matched samples across three methylation platforms: EPICv2, EPICv1 and WGBS (see Methods and Additional File 1: Table S13). Principal component analysis (Figs. 5a-c) of the common 586,916 CpG sites reveals very similar data projections for both EPICv1 and EPICv2, with only a difference of 0.09% in variance explained, while the variance explained by WGBS differs by over 10% from the arrays. Using the row-linear method from the Bioconductor package *consensus* [48], we calculated sensitivity (the platform-wise regression slope from the consensus fit) and precision (the platform-wise residual scatter around the regression line; noting that smaller values are superior) for the 586,916 probes (Figs. 5d,e). The difference between EPICv2 and EPICv1 is negligible for both sensitivity to methylation change (paired Cohen’s* d* = 0.14) and measurement precision (paired *d* = 0.03). WGBS is the clear outlier of the three platforms, with large advantages in sensitivity over arrays but inferior precision (*d* > 1.4, all comparisons with arrays). The gain in sensitivity from WGBS occurs principally towards the extremes of the methylation distribution (Additional File 6: Figure S12a). Pairwise comparison of methylation averages between the three platforms likewise reveals a correlation coefficient of 1.00 between arrays and 0.97 each when each array is compared to WGBS (Additional File 6: Figure S12b). Within both array platforms, we observed a small increase in sensitivity in Infinium Type II probes compared to Type I, coupled with a similar sized loss of precision (Figs. 5f,g). The sensitivity difference may be explained by the fact that Type I probe designs assume a completely methylated or unmethylated epitype across the 50 bp probe hybridisation site, potentially reducing their binding affinity in regions of local methylation heterogeneity.

### Cross-reactivity of EPICv2 probes

**Fig. 6:**
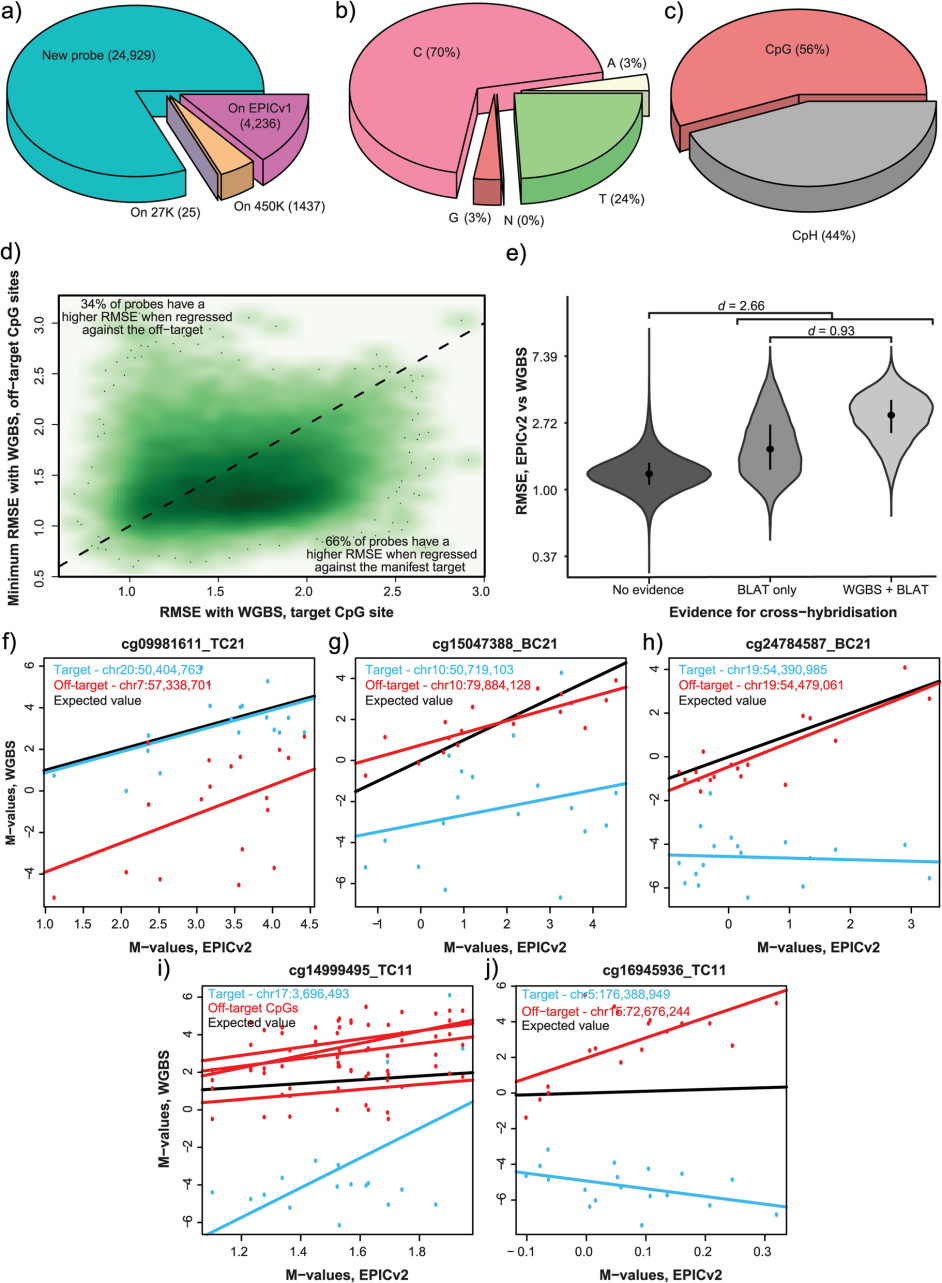
Cross-reactivity of EPICv2 probes. **a**) Proportion of cross-hybridising EPICv2 probes targeting cytosines present on EPICv2 or previous versions of the Illumina methylation array. Number denotes the platform on which the probe first appeared. **b**) Composition of predicted off-target nucleotides for all cross-hybridising cg probes. **c**) Dinucleotide composition for all off-target cytosines. **d**) Scatterplot of root mean square errors (RMSE) between the methylation of 17,928 EPICv2 probes and matched WGBS methylation for the corresponding on-target CpG (x-axis), and between that probe and its most similar off-target CpG (y-axis). **e**) Root mean squared error (RMSE) between methylation of EPICv2 probes and their matched on-target WGBS methylation, grouped by evidence for cross-hybridisation. **f**-**j**) Scatterplots of EPICv2 M-values against matched WGBS methylation from Illumina manifest MAPINFO on-target (blue) and off-target CpGs (red) for five selected EPICv2 probes, with IlmnID in title. Black line represents the expected value of WGBS given EPICv2

The cross-hybridisation of Infinium probes to off-target genomic regions is a known pitfall affecting previous versions of Illumina methylation arrays [16, 23, 27, 28]. Cross-hybridisation can interfere with the true methylation signal from the target site, and as such, inclusion of these probes in analysis risks spurious biological inference. To identify such probes, we used the BLAT local alignment tool to assess the degree of potential cross-hybridisation of all EPICv2 probes to genomic regions other than the stated target nucleotide in the Illumina manifest (‘off-target’). After BLAT probes with no homology to the reference genome (*n* = 24), Additional File 1: Table S15) or no homology to the stated MAPINFO site (*n* = 18), Additional File 1: Table S16) were removed. We report a total of 30,693 cross-hybridising probes, whose sequences confer 4,196,140 off-target in silico hybridisation events (Additional File 8), with a median of seven off-target alignments per probe (histogram of alignments per probe shown in Additional File 6: Figure S13). These 30,693 probes are identified under the “CH_BLAT” field in our new version of the manifest file (Additional File 4). If we include probes with no official mapping in the Illumina manifest (i.e. those labelled as “chr0” under the CHR field), of which 97% (6653/6889) are multimappers, the total number of cross-hybridising probes increases to 37,346. 30,627 of the 30,693 probes have cytosine targets, of which 80% are novel to EPICv2 (Fig. 6a), meaning that the number of cross-hybridising events is even more common on EPICv2 than its predecessor EPICv1 (which had only 1,645,024 off-target in silico hybridisation events from 32,738 cross-hybridising probes) [23]. This is despite the majority of cross-hybridising probes on EPICv1 having been excluded from EPICv2. Instead of simply reporting the 50 bp BLAT off-target hit as per our previous study [23], we have elected to report the putative off-target nucleotide that would be interrogated at the site of cross-hybridisation to resolve the methylation signal. We found 70% of the off-target nucleotides inferred from alignments from cg probes are cytosines (Fig. 6b), with 44% of these being CpH sites (Fig. 6c), which may lead to a bias towards lower methylation measurements. The remaining 30% of off-target sites are predominantly thymines, which may likewise bias the signal, since thymines are indistinguishable from unmethylated cytosine after bisulphite conversion.

The single-nucleotide resolution of our cross-hybridisation analysis, combined with the 18 matched WGBS samples used in the cross-platform analysis, allowed us to examine the specificity of 17,928 in silico cross-hybridising probes to their intended CpG target (see dropout thresholds in Methods). For these probes we quantified the degree of similarity of the EPICv2 methylation signal with both its corresponding on-target, and off-target WGBS methylation levels using root mean squared error (RMSE). We found approximately two-thirds of probes returned a lower RMSE with at least one of its off-target CpG sites than its intended target, suggesting more hybridisation to the off-target site (Fig. 6d). These probes are indicated in the “CH_WGBS_evidence” field of our new version of the manifest (Additional File 4), along with the total number of off-targets in “Num_offtargets”. The chromosomal position of the off-target site with the lowest RMSE with WGBS data is included in the field “Suggested_offtarget”. In general, in silico cross-hybridising probes have larger differences in methylation to their matched on-target WGBS measurements than probes with no BLAT evidence of cross-hybridisation (Fig. 6e, Cohen’s *d* = 2.66). Furthermore, those in silico cross-hybridizing probes with additional evidence for cross-hybridisation from WGBS are more divergent than those without (*d* = 0.93).

To illustrate the varying patterns of inferred probe cross-hybridisation we show five examples of new EPICv2 ‘cg’ probes, with their matched WGBS methylation measurements in Fig. 6. First, cg09981611_TC21 (Fig. 6f), which maps to both its on-target site on chromosome 20 and one off-target site on chromosome 7, shows a high concordance between EPICv2 methylation and WGBS methylation at the on-target site on chromosome 20. The methylation levels of cg15047388_BC21 (Fig. 6g), however, display a much closer agreement to its off-target site than the on-target site stated in the manifest. Both targets show some correlation, as demonstrated by the parallel slopes, suggesting a small degree of on-target hybridisation. In contrast the WGBS of the target site of cg24784587_BC21 (Fig. 6h) is uniformly unmethylated, yet the EPICv2 measured methylation shows high concordance with the WGBS at the more variably and highly methylated off-target, suggesting negligible hybridisation to the on-target site. Type I probe cg14999495_TC11 (Fig. 6i) has methylation levels similar to four distinct, methylated CpG off-target sites, whilst the methylation at its matched on-target WGBS site is predominantly unmethylated, again suggesting negligible hybridisation to the on-target site. Lastly, the invariant and intermediate EPICv2 methylation levels at cg16945936_TC11 (Fig. 6j) bisect its WGBS unmethylated on-target and methylated off-target site, suggestive of competitive probe hybridisation.

### Probe replicate comparison

**Fig. 7:**
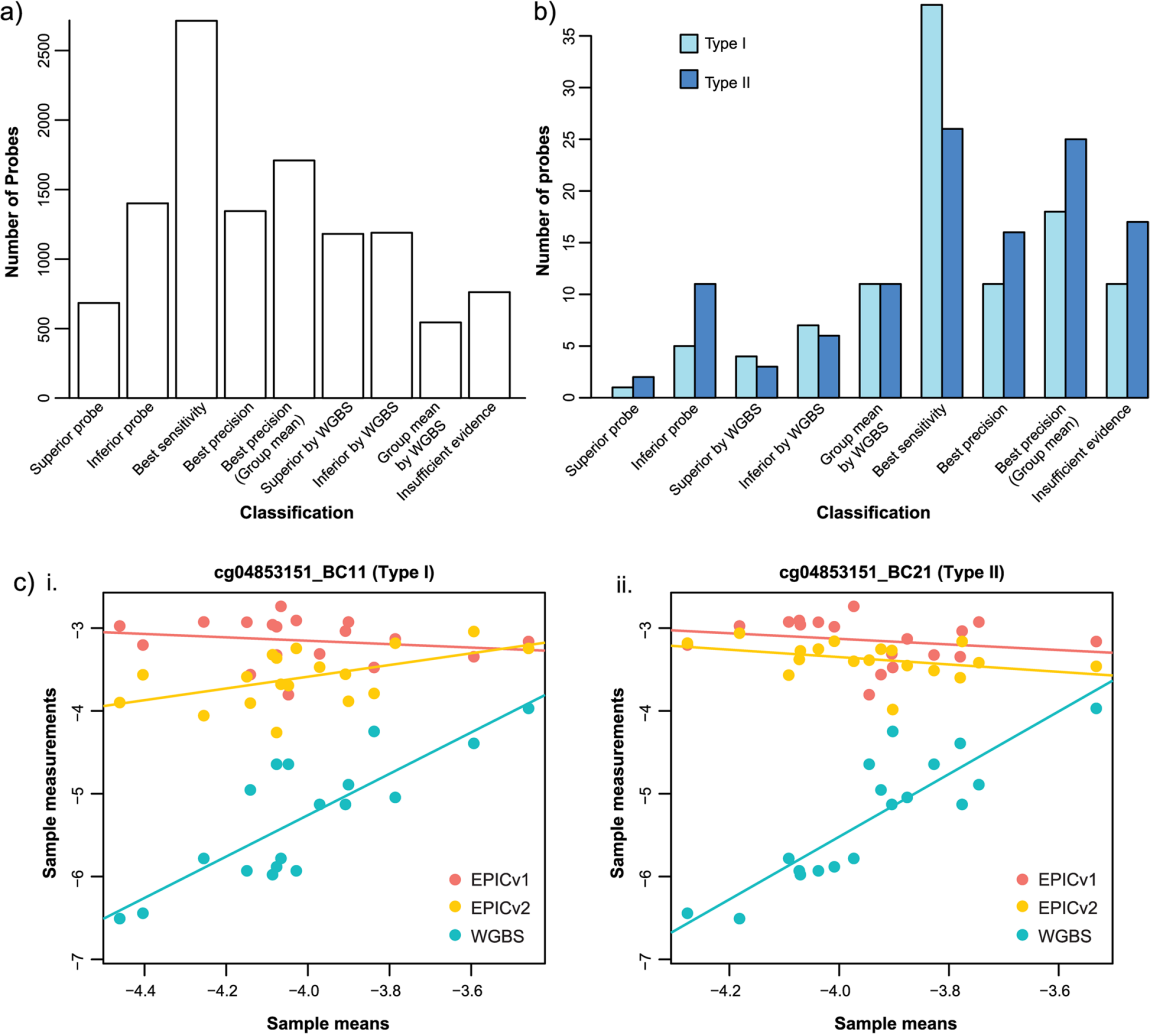
Comparison of replicate probes. **a**) Barplot of results from competitive comparison of probe replicates, where sets are grouped by target chromosomal position. **b**) Barplot of results from competitive comparison of probe replicates, for only the replicate sets that contain both Type I and Type II probes. **c**) Two row-linear fits of probes i) cg04853151_BC11 and ii) cg04853151_BC21. Note the more positive slope for EPICv2 on cg04853151_BC11 indicates greater sensitivity to methylation change than both the EPICv2 Type II probe in cii) and the EPICv2 Type II probe in ci.)

As described above, a subset of EPICv2 probes are replicated in the manifest by name, sequence and/or genomic location. These probes will present difficulties for researchers attempting to analyse EPICv2 data using existing packages, which currently assume that all probe locations within an array are unique. From our matched cross-platform data, we conducted analyses to compare replicates within probe sets to establish their concordance and recommend the most accurate probes within each set for analysis (subject to dropout thresholds and data availability, see Methods). Where both matched EPICv1 and WGBS data was available for the given probe set, the *consensus* package [48] was used to determine the superior probe with respect to sensitivity and/or precision. Where matched EPICv1 data was unavailable, minimum RMSE with matched WGBS within each group was used to determine the superior probe. Since it is possible that the sample mean of a probe set is more precise than any of its constituent probes, the sample-wise mean M-value for each probe set was also tested for superior precision where EPICv1 was available, and superior concordance with WGBS (via RMSE) when not. The results can be found under the fields “Rep_results_by_[NAME|SEQUENCE|LOCATION]” in our new version of the manifest (Additional File 4). Performance classifications (see Methods: Competitive Evaluation of Replicates) for the 11,529 location replicates are shown in Fig. 7a, and in tabular format for both probe counts and probe set counts in Additional File 1: Table S17. We found 36% of probe sets (1,865) had an undisputed superior probe (either by row-linear method or RMSE with WGBS), 32% (1,640) conferred best precision by probe set mean, 26% (1,345) superior sensitivity and precision divided between different probes in the set, and 6% (341) had insufficient evidence to make a call. Similar proportions were observed when replicate probes were grouped into probe sets by name and sequence (Additional File 6: Figures S14a,b). The choice of which probe(s) to select for analysis depends on the performance classification of the probe set, therefore we recommend a computational filtering strategy based on these results prior to downstream analysis.

Where location-replicate probe sets contained a mixture of Infinium Type I and Type II probes, the Type I probes showed a greater preference for sensitivity over precision (Fig. 7b), despite their assumed uniform epitype across the sequence. An example of their increased sensitivity is shown by the row-linear method applied to the pair of EPICv2 probes targeting the CpG locus cg04853151, Type I: cg04853151_BC11 (Fig. 7ci) and Type II: cg04853151_BC21 (Fig. 7cii). The EPICv2 Type II probe cg04853151_BC21 has a slope near 0 indicating that it is invariant to methylation change, like its matched Type I probe on EPICv1 (Fig. 7cii). In contrast, EPICv2 Type I probe cg04853151_BC11 (Fig. 7ci) has a more positive slope, indicating that it is more sensitive to methylation change than the corresponding Type II probes from EPICv1 (Fig. 7ci&ii) or EPICv2 (Fig. 7cii).

## New updated EPICv2 manifest for informed data analysis

In summary, through the above in silico analysis of the Illumina and SeSAMe manifest data, as well as empirical cross-platform analysis of matched DNA samples, we have constructed a new updated version of the EPICv2 manifest to guide researchers in their analyses and/or development of new EPICv2 analytical tools (Additional File 4). Our updated manifest contains comprehensive details including: 1) discrepancies between Illumina and SeSAMe manifests; 2) replicate probes defined by overlapping name, sequence or location; 3) probe overlap between Infinium array versions, again defined by overlapping name, sequence or location; 4) in silico and empirical evidence of cross-reactive probes; and 5) recommendations of superior probe within replicate or cross-hybridising probe sets.

## Discussion

Overall, we find EPICv2 a worthy successor to the previous Infinium methylation microarrays. EPICv2 has a high degree of within-array reproducibility based on the analysis of technical replicates, in line with recent studies [57, 59], including those with lower DNA input levels than recommended by the manufacturer. We find that between-array reproducibility is high over matched target locations and samples on EPICv1 and WGBS. Probes with technical problems on EPICv1 have largely been excluded from EPICv2. The new content unique to EPICv2 extends the genomic coverage including in regions of biological interest such as enhancers. The main drawback is the addition of a new set of probes whose sequences are even more cross-reactive to non-target genomic regions than those removed. We have conducted a more thorough in silico analysis of these cross-hybridisation events than previous studies on EPICv2’s predecessors, pinpointing over four million off-target sites at single nucleotide resolution. Where possible we have evaluated the relative extent of cross-reactivity via comparison to matched WGBS assays, finding supporting evidence of cross-reactivity in approximately two-thirds of probes tested. As such, we recommend that, at the least, the 11,878 probes flagged with a “Y” in the “CH_WGBS_evidence” field of our manifest be filtered out prior to analysis or the recommendations of off-target site considered.

A novel feature of EPICv2 is the addition of a set of probes whose probe name, sequence and/or target genomic location are replicated within the array. This presents a dilemma to the researcher, who wants to know the most reliable probe for a given locus. The current available workflows for EPICv2 on github simply randomly remove or collapse duplicate probes (https://github.com/perishky/meffil/, https://github.com/schalkwyk/wateRmelon, https://github.com/jokergoo/IlluminaHumanMethylationEPICv2manifest) [15, 18, 53]. With this in mind, we have analysed a diverse set of primary and cultured samples to provide a recommendation in our new manifest as to the superior probe in each replicate probe set group, or recommendation to use the probe set mean. While we do not claim these recommendations apply to all tissues and biological conditions, they serve as a pilot for potentially larger and more diverse technical studies to be conducted in the future by the broader research community.

## Conclusions

Like its predecessor EPICv1, EPICv2 continues to offer an affordable and user-friendly platform for genome-wide methylation analysis. We anticipate the wide range of new fields in our new manifest will be useful to users, who wish to make an informed choice when selecting probes for analysis. In particular our in silico identification and characterisation of cross-hybridising probes provides options for a user to apply an informed filtering strategy. Our manifest also serves as a starting point for further technical comparisons and reproducibility studies. For example, users can conveniently check the presence, absence, or sequence change of EPICv2 probes on its predecessor platforms, and design their comparisons accordingly. This paves the way for integration of new EPICv2 data with important datasets generated on older versions of the arrays, as well as improved biological understanding gained from the novel genomic regions on EPICv2.

### Supplementary Information

**Additional file 1: Table S1.** Summary of probes on EPICv2, divided by probe type and Infinium design type. **Table S2.** Details of nv probes and their matched variant within the COSMIC census database. **Table S3.** Summary of control probes on EPICv2. **Table S4.** Discrepant probes between Illumina manifest and script to recompute sequence from Illumina manifest 'Forward sequence' and 'IlmnID'. **Table S5.** Summary of number of probes per a) exact-replicate, b) location-replicate and c) sequence-only-replicate probe set. **Table S6.** Examples of a) exact-replicate, b) location-replicate and c) sequence-only-replicate probe sets. **Table S7.** Lists of IlmnIDs for probes that have different types of replicate. **Table S8.** Lists of IlmnIDs for probes that have different types of replicate, grouped by probe set. **Table S9.** Matches between EPICv2 probes and probes on older versions of the microarray based on 1) probe name, 2) target location (hg38) and 3) probe sequence of sesame manifests. **Table S10.** Number of replicate probes within older arrays (excluding control probes). **Table S11.** Number and percentage of sites targeted on each chromosome for each probe category. **Table S12.** Distribution of probes relative to different genomic features. **Table S13.** Details of samples profiled on EPICv2. **Table S14.** Number of probes with detection p-value >0.05 per sample. **Table S15.** Probes with no BLAT hits. **Table S16.** BLAT hit locations for probes that do not map to their target location in the Illumina manifest. **Table S17.** Results of competitive evaluation of location replicates.

**Additional file 2.** Supplementary Note. A pdf containing additional technical information about EPICv2 probe design and details of replicate probe types.

**Additional file 3.** A .R file containing the script for recomputing probe sequences from 'Forward sequence' and 'IlmnID' information in the Illumina manifest.

**Additional file 4.** Augmented EPICv2 manifest. A .csv file containing a new version of the manifest with additional information to aid interpretation and analysis of data.

**Additional file 5.** A pdf containing explanation of headers in the new version of the manifest (Additional File 4).

**Additional file 6: Supplementary Figure 1.** Venn diagram showing overlap in a) SNP loci and b) CpH loci targeted by different versions of the Innium methylation microarray. Note although EPICv2 includes 65 ‘rs’ probes, 3 are replicates, therefore the ‘rs’ probes target 62 unique loci. **Supplementary Figure 2.** Percentage of probe sites per probe category overlapping specififc a) genic and b) CpG island contexts. **Supplementary Figure 3.** Percentage (bars) and number (labels) of targeted CpG islands that overlap the specfied number of probe sites on a) EPICv1 and b) EPICv2. **Supplementary Figure 4.** a) Number of probes per sample with detection p-value >0.05. Dashed line indicates 10% CpG site threshold at which samples typically removed from further analysis. b) Control SNP probes on the EPICv2 array correctly group samples by donor individual. **Supplementary Figure 5.** Density plot of methylation values for samples profiled on EPICv2. **Supplementary Figure 6.** Density plot of methylation values for samples profiled on EPICv2 split by a-d) Infinium probe design (Type I or II) and e-h) probe category (retained, reinstated or new). **Supplementary Figure 7.** Density plot of methylation values for samples profiled on EPICv2 split by both Infinium probe design and probe category (retained, reinstated or new). **Supplementary Figure 8.** Comparison of LNCaP technical replicates with varying DNA input levels showing a) number of probes with detection p-value >0.05, Relative Log Methylation plots of b) PrEC and c) LNCaP and d) correlation matrix of methylation values between technical replicates. **Supplementary Figure 9.** Correlation plots of methylation β-value of technical replicates with varying DNA input levels for PrEC a) 500ng, b) 250ng, c) 125ng, and LNCaP d) 500ng, e) 250ng, f) 125ng. **Supplementary Figure 10.** Genome browser image showing methylation of prostate cancer tissue and cell line samples (of varying DNA input levels) at new EPICv2 site at FANTOM5 prostate enhancer region. **Supplementary Figure 11.** Genomic copy number for LNCaP (using PrEC as a reference) at a range of DNA input levels: a) 500ng, b) 250ng and c) 125ng. **Supplementary Figure 12.** a) Sensitivity as a function of row-linear intercept (M-value methylation domain) for the three platforms tested. b) Pairwise joint distributions of platform averages across all CpG sites tested. **Supplementary Figure 13.** Barplot of probe counts by given number of in silico cross-hybridisation events via BLAT. Note log transform for both axes. **Supplementary Figure 14.** Barplot of results from competitive comparison of probe replicates, where sets are grouped by a) IlmnID and b) probe sequence.

**Additional file 7.** Supplementary Note. A pdf containing additional results about the genomic distribution of EPICv2 probes relative to chromosomal location.

**Additional file 8.** A csv file containing list of off-target cross-hybridisation sites for EPICv2 probes. Contains IlmnID, chromosome, single nucleotide position (MAPINFO), BLAT-aligned genome (forward or reverse methylated, unmethylated or both), BLAT score, off-target base and boolean for whether base is a CpG site.

## Data Availability

The data generated as part of this study are available at the Gene Expression Omnibus (GEO) under accession GSE240482. The new manifest data (Additional File 4) will also be available as an R package at https://bioconductor.org/packages/EPICv2manifest. The scripts used for data analysis are available at https://github.com/clark-lab/EPICv2.

## Abbreviations

CpG: Cytosine-Guanine
WGBS: Whole Genome Bisulphite Sequencing
DMR: Differentially Methylated Region
ddNTP: Dideoxynucleotide triphosphate
C: Cytosine
T: Thymine
β: Beta
27K array: HumanMethylation27 BeadChip
450K array: HumanMethylation450 BeadChip
EPICv1: HumanMethylationEPIC BeadChip
EPICv2: HumanMethylationEPIC v2.0 BeadChip
dbSNP: Single Nucleotide Polymorphism Database
SNV: Single Nucleotide Variant
TSS: Transcription Start Site
PDX: Patient Derived Xenograft
RMSE: Root Mean Squared Error
