# Supplementary material for: Characterisation and reproducibility of the HumanMethylationEPIC v2.0 BeadChip for DNA methylation profiling
Source: BMC Genomics. 2024 Mar 6;25:251. doi: 10.1186/s12864-024-10027-5 (PMC10916044; doi:10.1186/s12864-024-10027-5)
Supplement: Supplementary file 2 — Additional file 2. Supplementary Note. A pdf containing additional technical information about EPICv2 probe design and details of replicate probe types. [file 12864_2024_10027_MOESM2_ESM.pdf]

**Supplementary Note:** Explanation of EPICv2 probe name and design with examples, and details of replicate probe types

**Illumina probe 'Name'**

The Illumina probe *'Name'* is comprised of a two letter 'locus target identifier' followed by an eight-digit number [1]. The 'locus target identifier' can be one of 'cg', 'ch', 'rs' and 'nv':

- 'cg' probes measure cytosine methylation levels at CpG sites.
- 'ch' probes measure cytosine methylation levels at CpH sites (CpA, CpT or CpC).
- 'rs' probes measure genotype at the corresponding Single Nucleotide Polymorphism Database (dbSNP) rsID.
- 'nv' probes measure genotype at 'nucleotide variant' or 'new variant' loci, single Nucleotide Variants (SNVs) that do not have an rsID.

Each Illumina probe *'Name'* refers not to the probe itself, but to a specific 122-mer sequence in a consistent CpG loci reference database built by Illumina, similar to the dbSNP database [2]. In Illumina's CpG database, each CpG dinucleotide is assigned a CpG cluster ID based on the flanking sequences either side. Note, this CpG cluster ID is the same as the probe *'Name'* field in the Illumina manifest. Therefore, where probe *'Name'* differs between array versions, despite probes having the same sequence, this can be due to changes in the flanking sequence beyond the probe sequence which have necessitated the assignment of a new CpG cluster ID [2]. This can occur when the database updates, for example when changing from hg19 (used in the design of 450K probes) to hg38 sequences (used in the design of EPICv1 probes). Examples of this can be found by comparing columns in Supplementary Data A – 'K450probeID' versus 'K450seqmatch'.

It is likely that in earlier versions of the methylation array probe names were assigned to unique probe sequences or addresses rather than genomic locations, as for example in the 27K array manifest there are a small number of probes that target the same CpG loci, each with a different probe name e.g. cg20657421 and cg00896220 both targeting CpG loci chr17: 36103124-36103126 (hg38).

**Example of 122-mer sequence** - for *'Name'*/CpG cluster ID cg12981137 the target locus is a CpG site at chr10:129,467,311 (hg38), and the 122-mer sequence is this CpG site and the 60 bp sequences flanking either side. This is found in the Illumina manifest in the field *'Forward_sequence'*:

5'GTCCCGACGCCCGCAGGTCCTCGCGGTGCGCACCGTTTGCGACTTGGTGAGTGTCTGGGT[CG]CCTCGCTCCCGGAAGAGTGC
GGAGCTCTCCCTCGGGACGGTGGCAGCCTCGAGTGGTCCT 3'

**Explanation of how strandedness is assigned to 122-mer sequence**
The top or bottom strand in the Illumina manifest field *'Strand_TB'* does not refer to the plus and minus strand in the reference genome database as may be expected. Instead the strand is assigned by Illumina for each sequence fragment individually based on a sequence walking method, as described in a technical note from Illumina [2]. Briefly, nucleotides either side of the CpG locus are counted outwards towards the 5' end of the sequence as n-1, n-2, n-3, etc., and outwards towards the 3' end of the sequence as n+1, n+2 n+3, etc, with equidistant bases forming a 'pair' (e.g. pair n+1 and n-1). The pairs are screened to identify the closest 'unambiguous pair' to the CpG locus. An unambiguous pair is defined as an A or T on one side of the CpG locus with its paired base on the other side of the CpG locus either a C or G (i.e. A/C, A/G, T/C, T/G). If the A or T of the first unambiguous pair is on the 5' side of the CpG locus then the *'Forward sequence'* is designated 'Top', whereas if it is on the 3' side then the *'Forward sequence'* is designated 'Bottom'. Thus each 122-mer *'Forward sequence'* in the Illumina manifest can either be 1) 'Forward, top', with its reverse complement designated 'Reverse, bottom', or 2) 'Forward, bottom' with its reverse complement designated 'Reverse, top'.

In the following example the first 'unambiguous pair' of bases is at n-2 and n-2 from the CpG at n, with an A at n-2 and a G at n+2. As the 'A/T' in the unambiguous pair ('A' underlined) is 5' of the CpG, this sequence is designated the 'Top' strand.

n-3 n-2 n-1 [ n ] n+1 n+2 n+3

5'… A G G C T C A C [C G] C G C A G T C C …3'

**Example of assigning strandedness**

cg12981137 *'Forward sequence'*
5'GTCCCGACGCCCGCAGGTCCTCGCGGTGCGCACCGTTTGCGACTTGGTGAGTGTCTGGGT[CG]CCTCGCTCCCGGAAGAGTGC
GGAGCTCTCCCTCGGGACGGTGGCAGCCTCGAGTGGTCCT 3'

In this example, the first unambiguous pair occurs at 1bp from CpG locus, with a 'T' at n-1 and 'C' at n+1. The A/T of the first unambiguous pair is a T on the 5' side of the CpG locus. Therefore this 'Forward sequence' is designated as the 'Top' strand. Therefore for this locus, there are two possible 'Design Strands' for probe design, 'forward-top', or the reverse strand: 'reverse-bottom'.

cg12981137 'Forward-top' with reverse strand:
Forward top
5'GTCCCGACGCCCGCAGGTCCTCGCGGTGCGCACCGTTTGCGACTTGGTGAGTGTCTGGGT[CG]CCTCGCTCCCGGAAGAGTGC
GGAGCTCTCCCTCGGGACGGTGGCAGCCTCGAGTGGTCCT 3'

Reverse bottom
3'CAGGGCTGCGGGCGTCCAGGAGCGCCACGCGTGGCAAACGCTGAACCACTCACAGACCCA[GC]GGAGCGAGGGCCTTCTCAC
GCCTCGAGAGGGAGCCCTGCCACCGTCGGAGCTCACCAGGA 5'

For probe design on the 'reverse-bottom' strand, it is oriented in a 5' to 3' direction

cg12981137 'Reverse-bottom':
Reverse bottom, reverse complemented
5'AGGACCACTCGAGGCTGCCACCGTCCCGAGGGAGAGCTCCGCACTCTTCCGGGAGCGAGG[CG]ACCCAGACACTCACCAAGTC
GCAAACGGTGCGCACCGCGAGGACCTGCGGGCGTCGGGAC 3'

**Explaining the relationship between 'Forward_sequence', the 4 character IlmnID suffix and probe sequence**

Each row of the Illumina manifest provides the details for a unique probe, including design details. The columns *'Strand_FR'* and *'Strand_TB'* inform which strand of the 122-mer *'Forward sequence'* was used to design the probe. For example, a 122-mer designated as 'Forward, top' could have a probe designed on either the 'Forward, top' (*'Strand_FR'* = F, *'Strand_TB'* = T) or 'Reverse, bottom' strand (*'Strand_FR'* = R, *'Strand_TB'* = B). Whereas a 122-mer designated as 'Forward, bottom' could have a probe designed on either the 'Forward, bottom' (*'Strand_FR'* = F, *'Strand_TB'* = B) or 'Reverse, top' (*'Strand_FR'* = R, *'Strand_TB'* = T) strand. For example, the CpG locus 'cg12981137' is targeted by four probes, two of which are designed on the 'Forward, top' strand (cg12981137_TC11, cg12981137_TC21), and two of which are designed on the 'Reverse, bottom' strand (cg12981137_BO11, cg12981137_BC21). Whereas 'cg03254865' has 3 probes, one of which is designed on the 'Forward, bottom' strand (cg03254865_BO11), and two of which are designed on the 'Reverse, top' (cg03254865_TC21, cg03254865_TC22). For each probe the strand used for design (the 'Design strand') is indicated not only by the *'Strand_FR'* and *'Strand_TB'* columns but also by the first letter of the cg identifier appendix in the *'IlmnID'* column, which can either be a T (top) or B (bottom). Additionally probes can be designed on the bisulfite converted DNA itself 'Converted', or on the 'Opposite' strand synthesized after whole genome amplification of bisulfite converted DNA. For each probe, whether the converted or opposite strand is used for probe design is indicated by either a C or O in the second letter of the cg identifier appendix in the *'IlmnID'* column and also in the *'Strand_CO'* column.

<u>Probe design steps</u>

With knowledge of the 'Design Strand' the probe sequence in manifest columns *'AlleleA_ProbeSeq'* and (Type I only) *'AlleleB_ProbeSeq'* can be reproduced through the following steps:

1) Orient the 'Design strand' in the 5' to 3' direction.

2) Take the reverse complement of the design strand in step 1) (reversal necessary as single base extension of the probes occurs at the 3' end of the probe).

3) Perform *in silico* bisulfite conversion of the reverse complement sequence from step 2), assuming a) methylated Cs at CpG loci and b) unmethylated Cs at CpG loci.

4) For **Type I** probes designed on the '**Converted**' strand (identified by 'C' in column *'Strand_CO'*) obtain the probe sequence by taking the reverse complement of the target CpG locus and 48bp sequence 3' of the CpG target loci, using the:

a) methylated CpG *in silico* bisulfite converted DNA from step 3a for a methylated probe

b) unmethylated CpG *in silico* bisulfite converted DNA from step 3b for an unmethylated probe

Note: In both methylated and unmethylated Type I probe designs the probe extension site will be the base immediately 5' of the target CpG locus. The expected nucleotide is recorded in manifest column *'Next_Base'* with A and T labeled with red dye and C and G labeled with green.

5) For **Type II** probes designed on the '**Converted**' strand ('C' in column *'Strand_CO'*) obtain the probe sequence by taking the reverse complement of the G of the target CpG locus and 49bp sequence 3' of the CpG target loci, using the methylated CpG *in silico* bisulfite converted DNA from step 3a. Replace all CGs with CR: R is an unspecified purine base (A or G) allowing binding to both unmethylated Cs converted to Ts and methylated Cs, thus Type II probes are agnostic to CpG methylation status within probe bodies. Additionally, if the base at the 5' end of a Type II probe hybridizes to the C of a CpG on the converted strand, convert the probe base to R.

Note: In Type II probes the probe extension site will be the cytosine of the target CpG locus, with an A (red) annealing binding at an unmethylated cytosine, and G (green) at a methylated cytosine.

For detailed explanation of Type I and II probes see Pidsley and colleagues 2016 [3]

6) For probes designed on the '**Opposite**' strand ('O' in column *'Strand_CO'*) skip step 2 but then take the reverse complement of the *in silico* bisulfite converted strand from step 3a/b to get the opposite strand. Then repeat steps 4a and 4b for Type I probe design or step 5 for Type II probes.

7) If the probe is a 'ch' probe then follow rules 1-6 above, but additionally:

a) if the 'ch' probe '*Strand_FR'* = R then replace the letter resulting 'D' (From the reverse complement of the 'H' with a 'T'.

b) if the 'ch' probe '*Strand_FR'* = F then shift the probe by 1bp towards the 3' end of the probe.

**Example of relationship between 'Forward_sequence', the 4 character IlmnID suffix and the probe sequence following above 'Probe design steps'**

*Probe design on a 122-mer sequence designated as 'Forward-top and Reverse-bottom'*

cg12981137 at chr10:129,467,311 (hg38)

Fragment of 122-mer 'Forward sequence' for cg12981137:

5' ...GTGAGTGTCTGGGT[CG]CCTCGCTCCCGGAA... 3'

Reverse strand of fragment of 122-mer 'Forward sequence' for cg12981137:

5' ...TTCCGGGAGCGAGG[CG]ACCCAGACACTCAC... 3'

Designation of top/bottom strandedness:

Forward sequence designated the top sequence:

Forward-top: 5' ...GTGAGTGTCTGGGT[CG]CCTCGCTCCCGGAA... 3'

Reverse sequence designated the bottom sequence:

Reverse-bottom: 5' ...TTCCGGGAGCGAGG[CG]ACCCAGACACTCAC... 3'

## A) Probe design on forward-top converted Type I (indicated by 'TC1' on IlmnID):

1) Forward-top sequence

5' …GTGAGTGTCTGGGT[CG]CCTCGCTCCCGGAA… 3'

2) Reverse complement of forward-top sequence

5' …TTCCGGGAGCGAGG[CG]ACCCAGACACTCAC… 3'

3) Bisulfite conversion (methylated CpGs)

5' …TTTCGGGAGCGAGG[CG]ATTTAGATATTTAT… 3'

Bisulfite conversion (unmethylated CpGs)

5' …TTTTGGGAGTGAGG[TG]ATTTAGATATTTAT… 3'

4) Hybridisation of probe specific to methylated CpGs

5' …TTTCGGGAGCGAGG[CG]ATTTAGATATTTAT… 3'
3' C[GC]TAAATCTATAAATA… 5'
cg12981137_TC11 (Type I probe B)

Hybridisation of probe specific to unmethylated CpGs

5' …TTTTGGGAGTGAGG[TG]ATTTAGATATTTAT… 3'
3' C[AC]TAAATCTATAAATA… 5'
cg12981137_TC11 (Type I probe A)

## B) Probe design on forward-top converted Type II (indicated by 'TC2' on IlmnID):

1) Forward-top sequence

5' …GTGAGTGTCTGGGT[CG]CCTCGCTCCCGGAA… 3'

2) Reverse complement of forward-top sequence

5' …TTCCGGGAGCGAGG[CG]ACCCAGACACTCAC… 3'

3) Bisulfite conversion (methylated CpGs)

5' …TTTCGGGAGCGAGG[CG]ATTTAGATATTTAT… 3'

Bisulfite conversion (unmethylated CpGs)

5' …TTTTGGGAGTGAGG[TG]ATTTAGATATTTAT… 3'

5) Hybridisation of probe specific to methylated CpGs

5' …TTTCGGGAGCGAGG[CG]ATTTAGATATTTAT… 3'
3' [GC]TAAATCTATAAATA… 5'
cg12981137_TC21 (Type II probe A)

Hybridisation of probe specific to unmethylated CpGs

5' …TTTTGGGAGTGAGG[TG]ATTTAGATATTTAT… 3'
3' [AC]TAAATCTATAAATA… 5'
cg12981137_TC21 (Type II probe B)

## C) Probe design on reverse-bottom converted Type II (indicated by 'BC2' on IlmnID):

1) Reverse-bottom sequence

5' …TTCCGGGAGCGAGG[CG]ACCCAGACACTCAC… 3'

2) Reverse complement of reverse-bottom sequence

5' …GTGAGTGTCTGGGT[CG]CCTCGCTCCCGGAA… 3'

3) Bisulfite conversion (methylated CpGs)

5' …GTGAGTGTCTGGGT[CG]TTTCGTTTTCGGAA… 3'

Bisulfite conversion (unmethylated CpGs)

5' …GTGAGTGTCTGGGT[TG]TTTTGTTTTTGGAA… 3'

5) Hybridisation of probe specific to methylated CpGs

5' …GTGAGTGTCTGGGT[CG]TTTCGTTTTCGGAA… 3'
3' [GC]AAARCAAAARCCTT… 5'
cg12981137_BC21 (Type II probe A)

Hybridisation of probe specific to unmethylated CpGs

5' …GTGAGTGTCTGGGT[TG]TTTTGTTTTTGGAA… 3'
3' [AC]AAARCAAAARCCTT… 5'
cg12981137_BC21 (Type II probe A)

D) Probe design on reverse-bottom opposite Type I (indicated by 'BO1' on IlmnID):

1) Reverse-bottom sequence

5' …TTCCGGGAGCGAGG[CG]ACCCAGACACTCAC… 3'

3) Bisulfite conversion (methylated CpGs)

5' …TTTCGGGAGCGAGG[CG]ATTTAGATATTTAT… 3'

Bisulfite conversion (unmethylated CpGs)

5' …TTTTGGGAGTGAGG[CG]ATTTAGATATTTAT… 3'

6) Opposite strand (reverse complement)

5' … ATAAATATCTAAAT[CG]CCTCGCTCCCGAAA … 3'

Opposite strand (reverse complement)

5' … ATAAATATCTAAAT[CA]CCTCACTCCCAAAA … 3'

4) Hybridisation of probe specific to methylated CpGs

5' … ATAAATATCTAAAT[CG]CCTCGCTCCCGAAA … 3'
3' [**G**C]GGAGCGAGGGCTTT… 5'
cg12981137_BO11 (Type I Probe B)

Hybridisation of probe specific to unmethylated CpGs

5' … ATAAATATCTAAAT[CA]CCTCACTCCCAAAA … 3'
3' [**G**T]GGAGTGAGGGTTTTT
cg12981137_BO11 (Type I Probe A)

*Note *'Next_base'* is incorrect in the Illumina manifest (as A), but correct in the Sesame manifest as *'nextBase'* (as G). In the Illumina manifest header description file it states that *'Next_base'* is the next base immediately after the target CpG site, rather than the next base after the probe end (the extension site). This means that the corresponding colour channel information *'Color_Channel'* and *'Col'* in the Illumina manifest is also incorrect for many Type 1 Opposite probes.

*<u>Probe design on a 122-mer sequence designated as 'Forward-bottom and Reverse-top'</u>*

## cg03254865 at chr5:71,494,301 (hg38)

## Fragment of 122-mer 'Forward sequence' for cg03254865:

5' …TGAAATGGTCAGTG[CG]TGGCTATCAGTTAT… 3'

## Reverse strand of fragment of 122-mer 'Forward sequence' for cg03254865:

5' …ATAACTGATAGCCA[CG]CACTGACCATTTCA… 3'

## Designation of top/bottom strandedness:

Forward sequence designated the bottom sequence:

Forward-bottom: 5' …TGAAATGGTCAGTG[CG]TGGCTATCAGTTAT… 3'

Reverse sequence designated the top sequence:

Reverse-top: 5' …ATAACTGATAGCCA[CG]CACTGACCATTTCA… 3'

A&B) Probe design on reverse-top converted Type II (indicated by 'TC2' on IlmnID):

1) Reverse-top sequence

5' …ATAACTGATAGCCA[CG]CACTGACCATTTCA… 3'

2) Reverse complement of reverse-top sequence

5' …TGAAATGGTCAGTG[CG]TGGCTATCAGTTAT… 3'

3) Bisulfite conversion (methylated CpGs)

5' …TGAAATGGTTAGTG[CG]TGGTTATTAGTTAT… 3'

Bisulfite conversion (unmethylated CpGs)

5' …TGAAATGGTTAGTG[TG]TGGTTATTAGTTAT… 3'

5) Hybridisation of probe specific to methylated CpGs

5' …TGAAATGGTTAGTG[CG]TGGTTATTAGTTAT… 3'
3' [GC]ACCAATAATCAATA… 5'
cg03254865_TC21 (Type II probe A)
3' [GC]ACCAATAATCAATA… 5'
cg03254865_TC22 (Type II probe A)

Hybridisation of probe specific to unmethylated CpGs

5' …TGAAATGGTTAGTG[TG]TGGTTATTAGTTAT… 3'
3' [AC]ACCAATAATCAATA… 5'
cg03254865_TC21 (Type II probe A)
3' [AC]ACCAATAATCAATA… 5'
cg03254865_TC22 (Type II probe A)

C) Probe design on forward-bottom opposite Type I (indicated by 'BO1' on IlmnID):

1) Forward-bottom sequence

5' …TGAAATGGTCAGTG[CG]TGGCTATCAGTTAT… 3'

3) Bisulfite conversion (methylated CpGs)

5' …TGAAATGGTTAGTG[CG]TGGTTATTAGTTAT… 3'

Bisulfite conversion (unmethylated CpGs)

5' …TGAAATGGTTAGTG[TG]TGGTTATTAGTTAT… 3'

6) Opposite strand (reverse complement)

5' …ATAACTAATAACCA[CG]CACTAACCATTTCA… 3'

Opposite strand (reverse complement)

5' …ATAACTAATAACCA[CA]CACTAACCATTTCA… 3'

4) Hybridisation of probe specific to methylated CpGs

5' …ATAACTAATAACCA[CG]CACTAACCATTTCA… 3'
3' [**G**C]GTGATTGGTAAAGT… 5'
cg03254865_BO11 (Type I probe B)

Hybridisation of probe specific to unmethylated CpGs

5' …ATAACTAATAACCA[CA]CACTAACCATTTCA… 3'
3' [**G**T]GTGATTGGTAAAGT… 5'
cg03254865_BO11 (Type I probe A)

*Note *'Next_base'* is incorrect in the Illumina manifest (as A), but correct in the Sesame manifest as *'nextBase'* (as G). In the Illumina manifest header description file it states that *'Next_base'* is the next base immediately after the target CpG site, rather than the next base after the probe end (the extension site). This means that the corresponding colour channel information *'Color_Channel'* and *'Col'* in the Illumina manifest is also incorrect for many Type 1 Opposite probes.

*Probe design on a 122-mer sequence designated as 'Reverse-top and Forward-bottom'*

## cg15879059 at chr11:579,119 (hg38)

## Fragment of 122-mer 'Forward sequence' for cg15879059:

5' …TGCTAGGATGACAGG[CG]TGAGCCACTGCACCT… 3'

## Reverse strand of fragment of 122-mer 'Forward sequence' for cg15879059:

5' … AGGTGCAGTGGCTCA[CG]CCTGTCATCCTAGCA … 3'

## Designation of top/bottom strandedness:

Forward sequence designated the bottom sequence:
Forward-bottom: 5' …TGCTAGGATGACAGG[CG]TGAGCCACTGCACCT… 3'

Reverse sequence designated the top sequence:
Reverse-top: 5' … AGGTGCAGTGGCTCA[CG]CCTGTCATCCTAGCA … 3'

A) Probe design on reverse-top opposite Type I (indicated by 'T01' on IlmnID):

1) Reverse-top sequence

5' … AGGTGCAGTGGCTCA[CG]CCTGTCATCCTAGCA … 3'

3) Bisulfite conversion (methylated CpGs)

5' … AGGTGTAGTGGTTTA[CG]TTTGTTATTTTAGTA …3'

Bisulfite conversion (unmethylated CpGs)

5' … AGGTGTAGTGGTTTA[TG]TTTGTTATTTTAGTA …3'

6) Opposite strand (reverse complement)

5' … TACTAAAATAACAAA[CG]TAAACCACTACACCT … 3'

Opposite strand (reverse complement)

5' … TACTAAAATAACAAA[CA]TAAACCACTACACCT … 3'

4) Hybridisation of probe specific to methylated CpGs

5' … TACTAAAATAACAAA[CG]TAAACCACTACACCT … 3'
3' [**G**C]ATTTGGTGATGTGGA… 5'
cg15879059_TO11 (Type I probe B)

Hybridisation of probe specific to unmethylated CpGs

5' … TACTAAAATAACAAA[CA]TAAACCACTACACCT … 3'
3' [**G**T]ATTTGGTGATGTGGA… 5'
cg15879059_TO11 (Type I probe A)

**Replicate probe types and relationship with IlmnID naming system**

There are different types of replicate probes on EPICv2. According to the EPICv2 Illumina manifest 'Rep_Num' field there are 5,141 probes, that each have between 2-10 replicates (**Additional File 1**: **Table S5a**). These probes are exact replicates, that is sets of probes with the same 'Name' (cgXXXXXXX) and the same probe sequence (**Additional File 1**: **Table S6**). These are exact replicates, meaning that sets of probes with the same 'Name' also have the same probe sequence, and consistent with this, their IlmnIDs only differ by the 4$^{th}$ letter of the suffix (e.g. cg09617579_BC11, cg09617579_BC12, cg09617579_BC13) (**Additional File 1**: **Table S6**). Due to their identical 'Name' and identical sequence we term these 'exact-replicates' (listed in **Additional File 1**: **Table S7**). Additionally, there are 112 locations, which surprisingly are each targeted by multiple (2 to 4) probes with the same 'Name', but different probe sequences. We term these probes 'location-replicates' (**Additional File 1**: **Table S5b, 6 & 7**). For example, there are four probes that share the probe 'name' 'cg12981137' but each has a distinct sequence, indicated within the first three characters of their IlmnID suffix (cg12981137_BO11, cg12981137_TC11, cg12981137_TC21, cg12981137_BC21). For each of these four probes the 4$^{th}$ character of the suffix is 1, confirming that they are not considered 'exact-replicates' of one another due to their different sequences (see **Figure 1** & **above** for explanation of the sequence differences between the probes of location-replicate cg12981137). Interestingly, we note that 28 CpG sites targeted by 'location-replicates' are also targeted by 'exact-replicates'. Lastly, there are an additional 1,000 probes that are replicates in terms of probe sequence (434 unique probe sequences), but not location or name, which we have termed 'sequence-only-replicates' (**Additional File 1**: **Table S5c, 6 & 7**). These are a subset of 6,889 probes in the manifest that are lacking chromosome mapping information for unknown reasons.

## References


1. Illumina: **Infinium MethylationEPIC v2.0 Manifest File Release Notes.** https://sapac.support.illumina.com/content/dam/illumina-support/documents/downloads/productfiles/methylationepic/MethylationEPIC%20v2%20Files.zip; Accessed 23rd December 2022.
2. Illumina: **CpG loci identification technical note.** https://www.illumina.com/content/dam/illumina/gcs/assembled-assets/marketing-literature/cpg-loci-identification-tech-note-m-gl-00921/cpg-loci-identification-tech-note-m-gl-00921.pdf; 2022.
3. Pidsley R, Zotenko E, Peters TJ, Lawrence MG, Risbridger GP, Molloy P, Van Djik S, Muhlhausler B, Stirzaker C, Clark SJ: **Critical evaluation of the Illumina MethylationEPIC BeadChip microarray for whole-genome DNA methylation profiling.** *Genome Biol* 2016, **17:**208.
