# Supplementary material for: Characterisation and reproducibility of the HumanMethylationEPIC v2.0 BeadChip for DNA methylation profiling
Source: BMC Genomics. 2024 Mar 6;25:251. doi: 10.1186/s12864-024-10027-5 (PMC10916044; doi:10.1186/s12864-024-10027-5)
Supplement: Supplementary file 5 — Additional file 5. A pdf containing explanation of headers in the new version of the manifest (Additional File 4). [file 12864_2024_10027_MOESM5_ESM.pdf]

Technical Note: Characterisation and reproducibility of the HumanMethylationEPIC v2.0 BeadChip for DNA methylation profiling

Heading descriptors for the additional columns added to EPICv2 manifest EPIC-8v2-0_A1.csv

CpG_chrm, **CpG_beg**, **CpG_end** – EPICv2 coordinates from Sesame manifest

MismatchPos – vector of probes where "Y" indicates discrepant genomic position between Sesame and Illumina, includes those that are missing mapping information in Illumina manifest (chr0)

MissingPos - vector of probes where "Y" indicates probes missing mapping information in Illumina manifest

namerep – vector of probes where "Y" indicates probes that have replicates based on probe name match (this vector can be married up with **Name** column to select a single probe to represent each name replicate)

seqrep – vector of probes where "Y" indicates probes that have exact sequence matches with other probes within EPICv2

seqrep_IlmnIDs - IlmnIDs of those probes with exact sequence matches with other probes within EPICv2 (corresponding to **seqrep**)

seqrep_RepNum – replicate number of those probes with exact sequence matches with other probes within EPICv2 (corresponding to **seqrep** and **seqrep_IlmnIDs**). Can be used to e.g. filter for only 1 probe per sequence-replicate.

posrep – vector of probes where "Y" indicates probes that have exact genomic position matches with other probes within EPICv2 (based on Illumina genomic positions – note, none of the affected probes are discrepant with Sesame mapping)

posrep_IlmnIDs - IlmnIDs of those probes with exact genomic position matches with other probes within EPICv2 (corresponding to **posrep**)

posrep_RepNum – replicate number of those probes with exact genomic position matches with other probes within EPICv2 (corresponding to **posrep** and **posrep_IlmnIDs**). Can be used to e.g. filter for only 1 probe per genomic-position-replicate.

EPICv1probeID – vector of EPICv1 probe names where probe names match between EPICv1 and EPICv2

EPICv1seqmatch - vector of EPICv1 probe names where probe sequences match between EPICv1 and EPICv2

EPICv1locmatch - vector of EPICv1 probe names where genomic locations match between EPICv1 and EPICv2 (based on Sesame locations)

K450probeID – vector of 450K probe names where probe names match between 450K and EPICv2

K450seqmatch - vector of 450K probe names where probe sequences match between 450K and EPICv2

K450locmatch - vector of 450K probe names where genomic locations match between 450K and EPICv2 (based on Sesame locations)

K450locmatch2 - vector of 450K probe names where additional 450K probes have a genomic location match between 450K and EPICv2 (based on Sesame locations)

K27probeID - vector of 27K probe names where probe names match between 27K and EPICv2

K27seqmatch - vector of 27K probe names where probe sequences match between 27K and EPICv2

K27locmatch - vector of 27K probe names where genomic locations match between 27K and EPICv2 (based on Sesame locations)

K27locmatch2 - vector of 27K probe names where additional 27K probes have a genomic location match between 27K and EPICv2 (based on Sesame locations)

CH_BLAT – vector of probes where "Y" indicates at least one *in silico* cross-hybridisation event (≥47 bp match) to a non-target region of the genome, predicted by BLAT. For a complete list of off-targets please see (Supplementary Table X).

CH_WGBS_evidence – subset vector of **CH_BLAT** where "Y" indicates a greater affinity for the off-target(s), via comparison to whole genome bisulphite sequencing on matched samples

RMSE_with_WGBS – root mean squared error when comparing probe methylation to matched target CpG site methylation from WGBS (M-values)

Num_offtargets – number of off-target *in silico* hybridisation events predicted by the probe sequence via BLAT

Suggested_offtarget – if **CH_WGBS_evidence** == "Y", the hg38 coordinate of the off-target cytosine conferring minimum RMSE with WGBS

Rep_results_by_NAME – results of competitive comparison between replicates, with replicate probe sets defined by column **Name**

Rep_results_by_SEQUENCE - results of competitive comparison between replicates, with replicate probe sets defined by column **seqrep_IlmnIDs**

Rep_results_by_LOCATION - results of competitive comparison between replicates, with replicate probe sets defined by column **posrep_IlmnIDs**
