# Supplementary material for: Characterisation and reproducibility of the HumanMethylationEPIC v2.0 BeadChip for DNA methylation profiling
Source: BMC Genomics. 2024 Mar 6;25:251. doi: 10.1186/s12864-024-10027-5 (PMC10916044; doi:10.1186/s12864-024-10027-5)
Supplement: Supplementary file 6 — Additional file 6: Supplementary Figure 1. Venn diagram showing overlap in a) SNP loci and b) CpH loci targeted by different versions of the Innium methylation microarray. Note although EPICv2 includes 65 ‘rs’ probes, 3 are replicates, therefore the ‘rs’ probes target 62 unique loci. Supplementary Figure 2. Percentage of probe sites per probe category overlapping specififc a) genic and b) CpG island contexts. Supplementary Figure 3. Percentage (bars) and number (labels) of targeted CpG islands that overlap the specfied number of probe sites on a) EPICv1 and b) EPICv2. Supplementary Figure 4. a) Number of probes per sample with detection p-value >0.05. Dashed line indicates 10% CpG site threshold at which samples typically removed from further analysis. b) Control SNP probes on the EPICv2 array correctly group samples by donor individual. Supplementary Figure 5. Density plot of methylation values for samples profiled on EPICv2. Supplementary Figure 6. Density plot of methylation values for samples profiled on EPICv2 split by a-d) Infinium probe design (Type I or II) and e-h) probe category (retained, reinstated or new). Supplementary Figure 7. Density plot of methylation values for samples profiled on EPICv2 split by both Infinium probe design and probe category (retained, reinstated or new). Supplementary Figure 8. Comparison of LNCaP technical replicates with varying DNA input levels showing a) number of probes with detection p-value >0.05, Relative Log Methylation plots of b) PrEC and c) LNCaP and d) correlation matrix of methylation values between technical replicates. Supplementary Figure 9. Correlation plots of methylation β-value of technical replicates with varying DNA input levels for PrEC a) 500ng, b) 250ng, c) 125ng, and LNCaP d) 500ng, e) 250ng, f) 125ng. Supplementary Figure 10. Genome browser image showing methylation of prostate cancer tissue and cell line samples (of varying DNA input levels) at new EPICv2 site at FANTOM5 prostate enhancer region. Sup [file 12864_2024_10027_MOESM6_ESM.pdf]

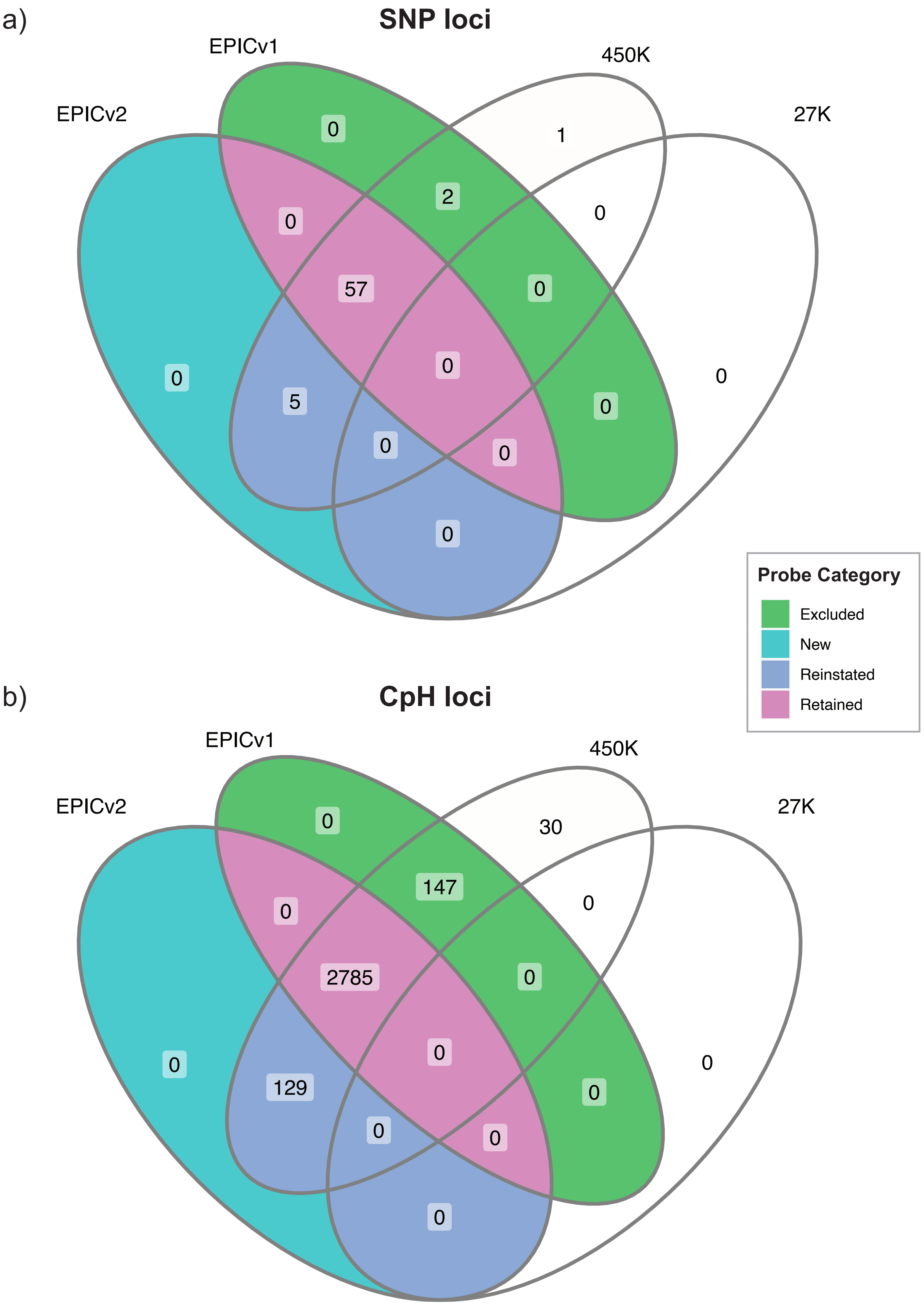

Supplementary Figure 1) Venn diagram showing overlap in a) SNP loci and b) CpH loci targeted by different versions of the Infinium methylation microarray. Note although EPICv2 includes 65 'rs' probes, 3 are replicates, therefore the 'rs' probes target 62 unique loci.

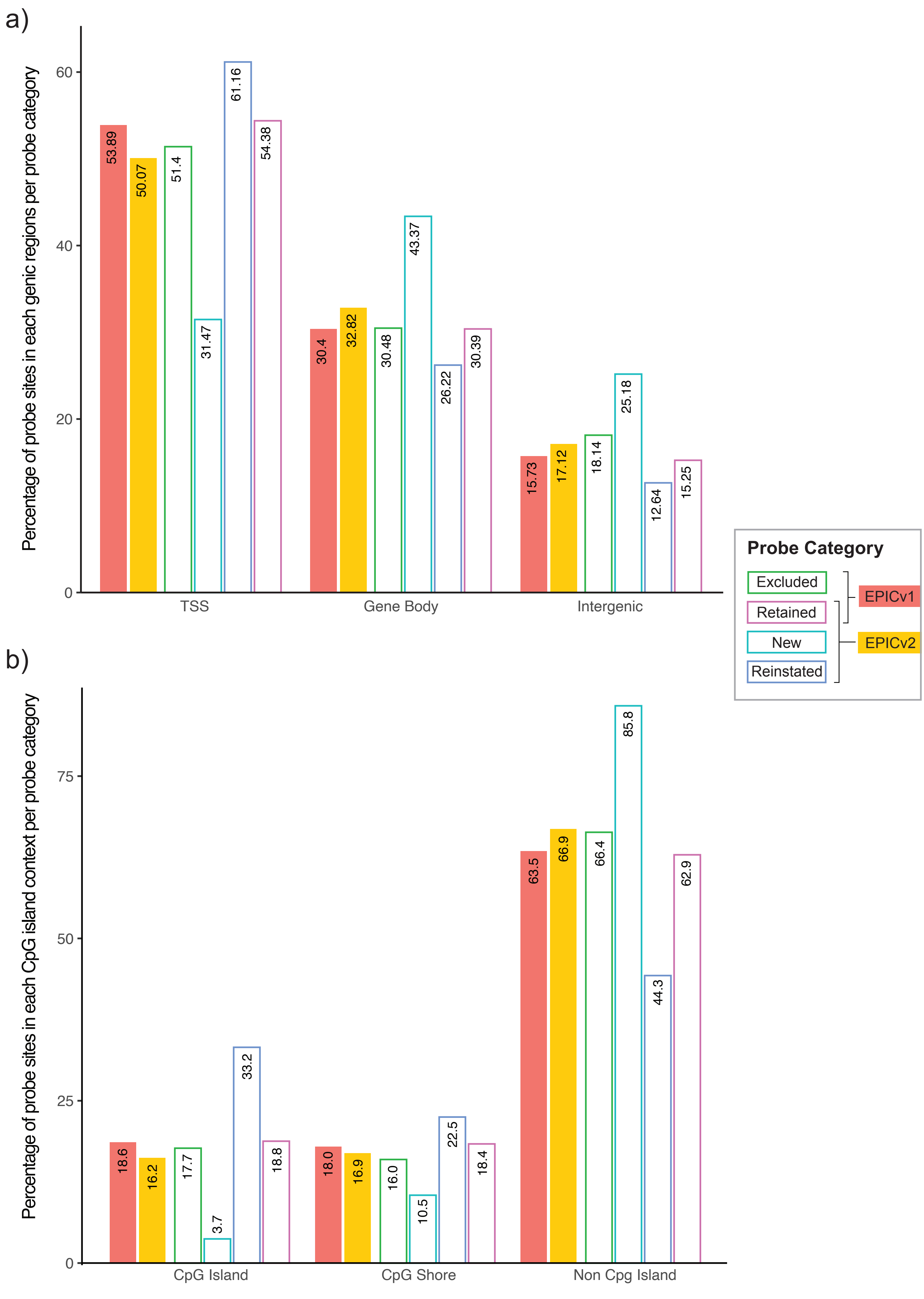

Supplementary Figure 2) Percentage of probe sites per probe category overlapping specific a) genic and b) CpG island contexts.

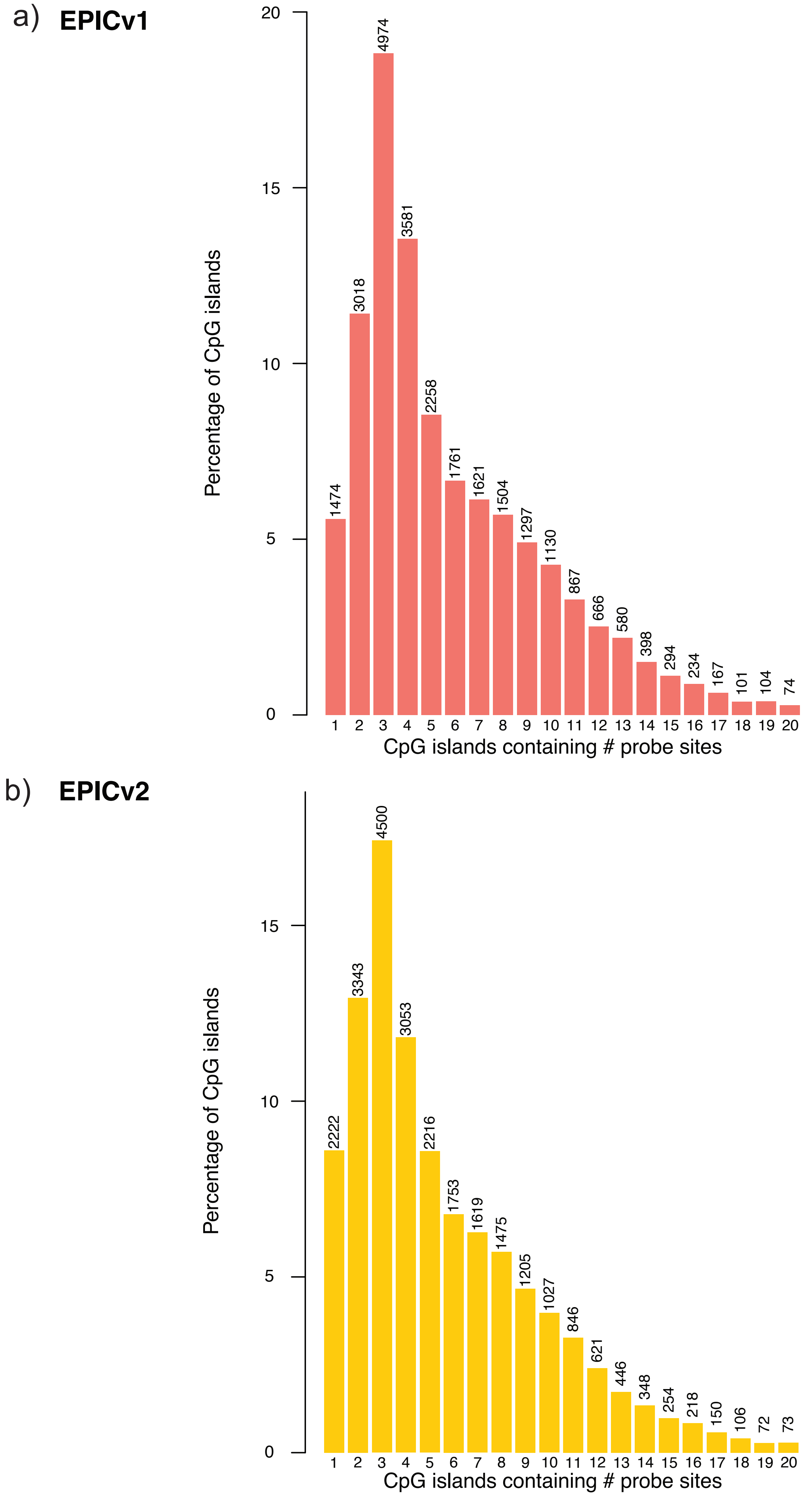

Supplementary Figure 3) Percentage (bars) and number (labels) of targeted CpG islands that overlap the specified number of probe sites on a) EPICv1 and b) EPICv2.

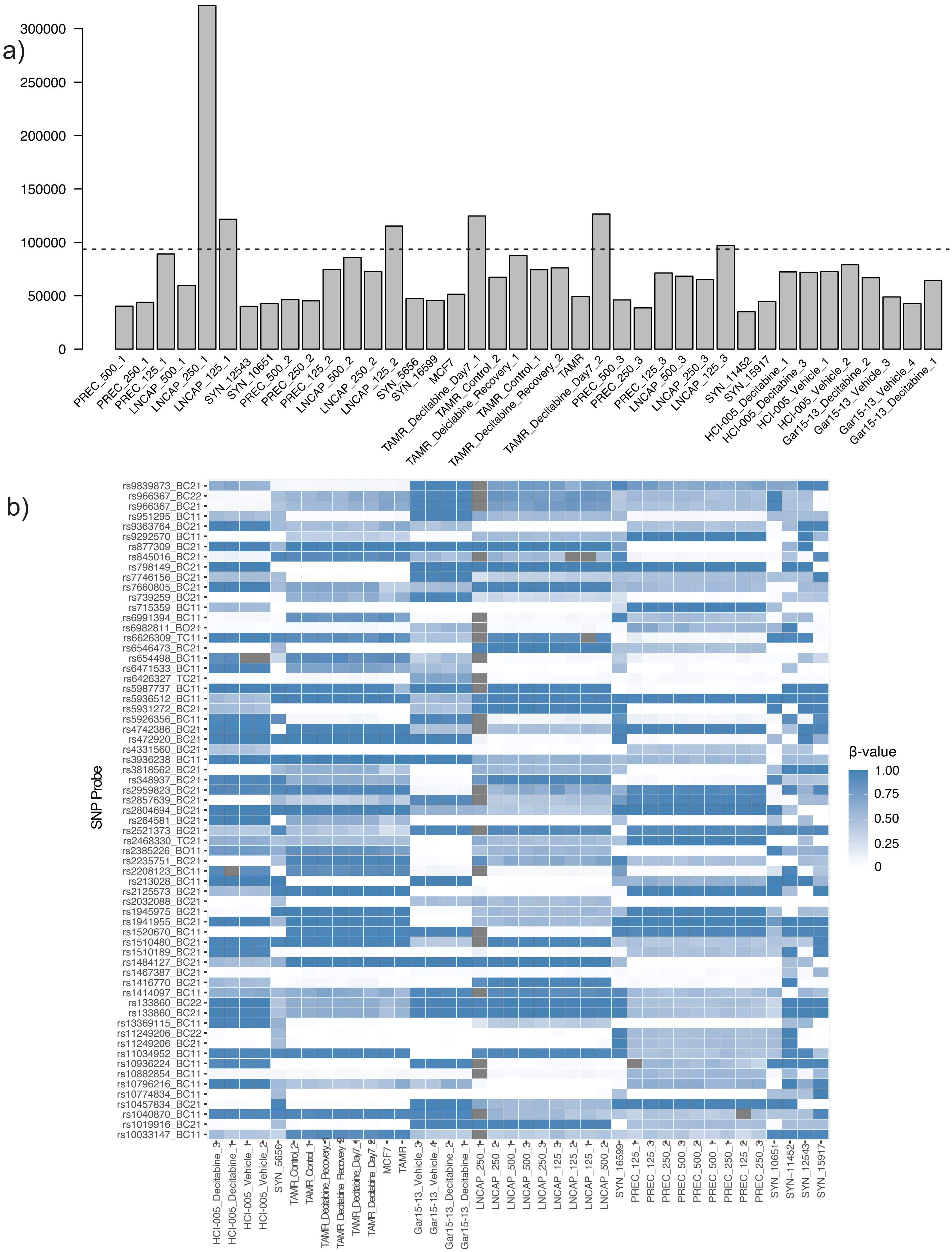

Supplementary Figure 4) a) Number of probes per sample with detection p-value >0.05. Dashed line indicates 10% CpG site threshold at which samples typically removed from further analysis. b) Control SNP probes on the EPICv2 array correctly group samples by donor individual .

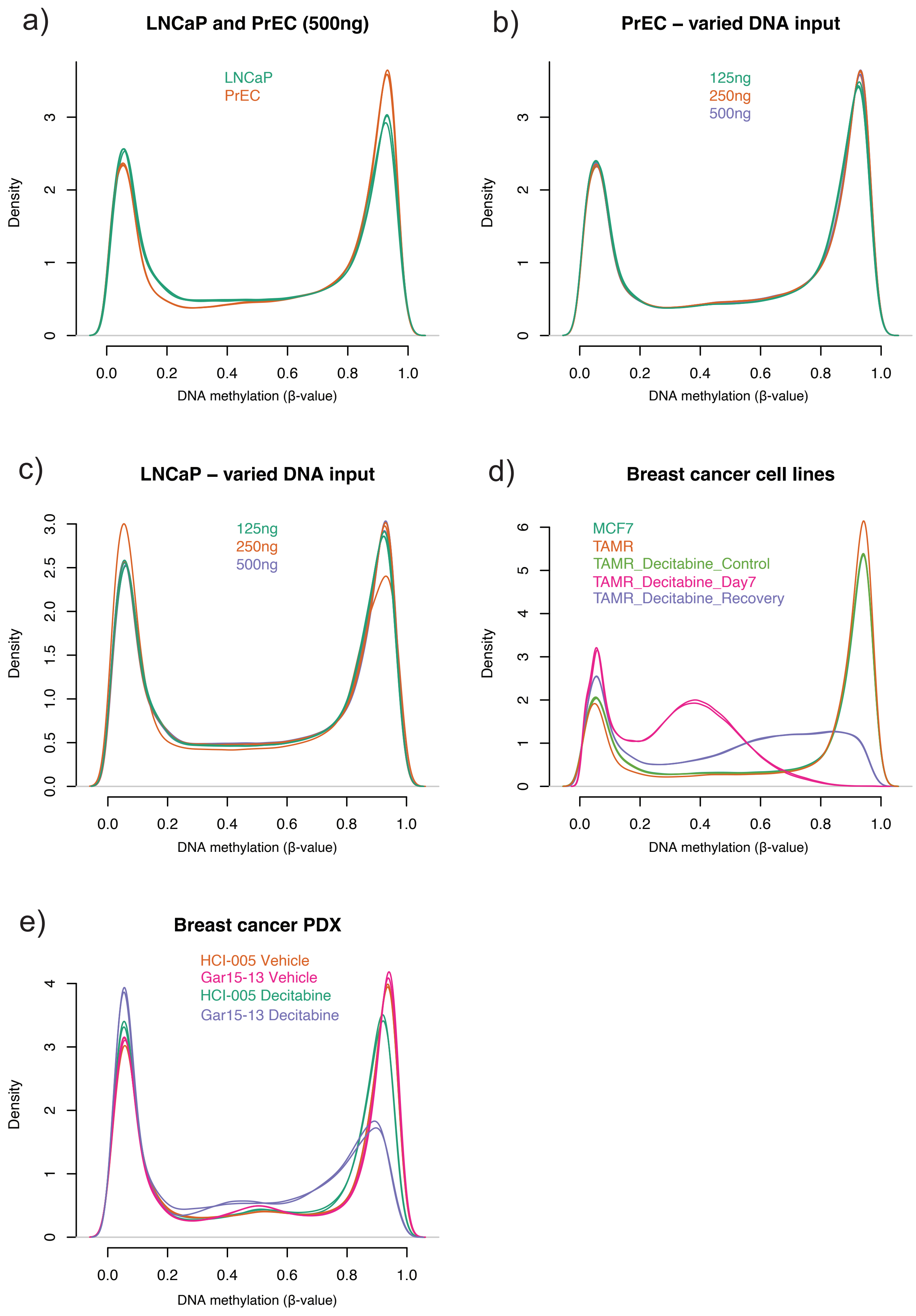

Supplementary Figure 5) Density plot of methylation values for samples profiled on EPICv2.

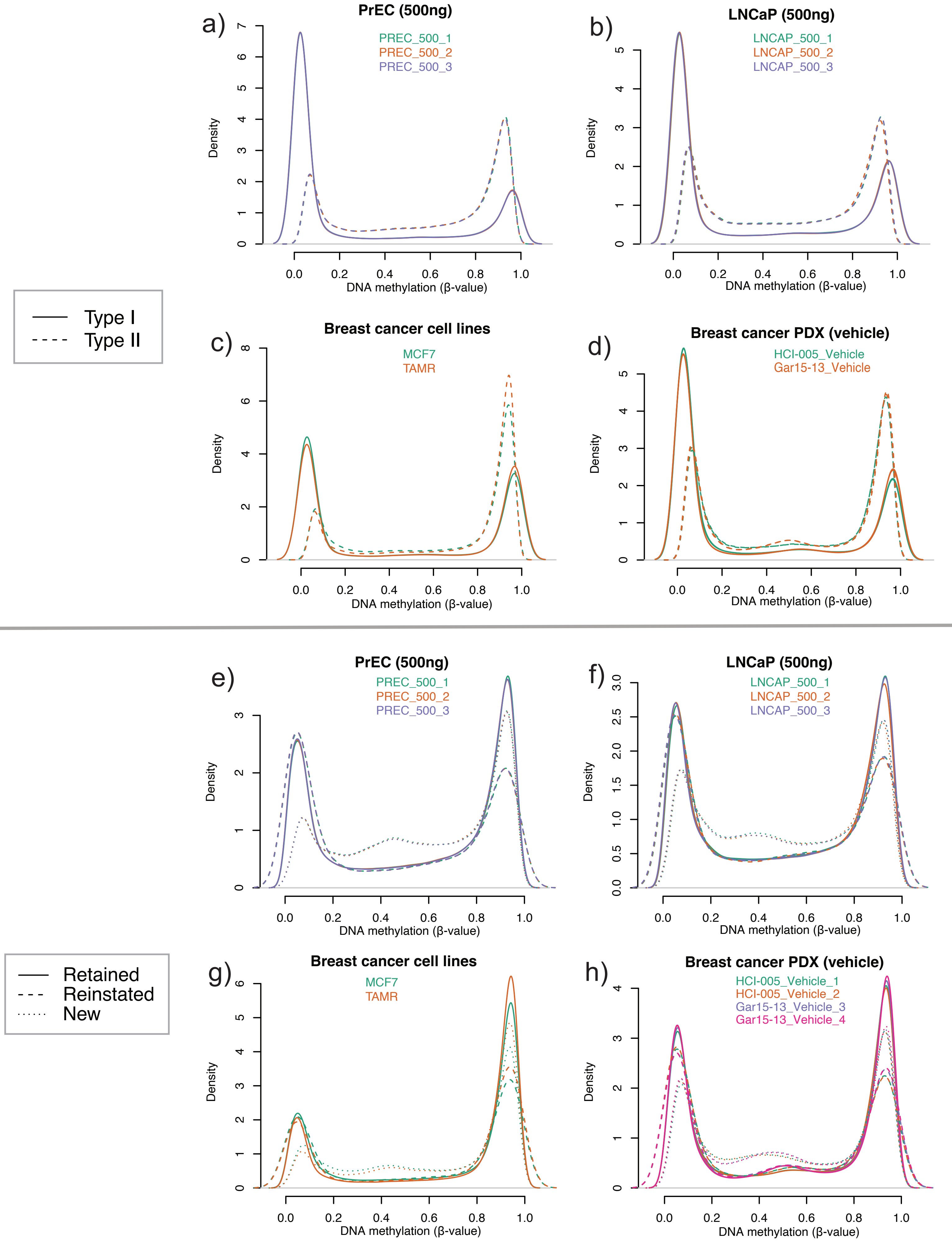

Supplementary Figure 6) Density plot of methylation values for samples profiled on EPICv2 split by a-d) Infinium probe design (Type I or II) and e-h) probe category (retained, reinstated or new).

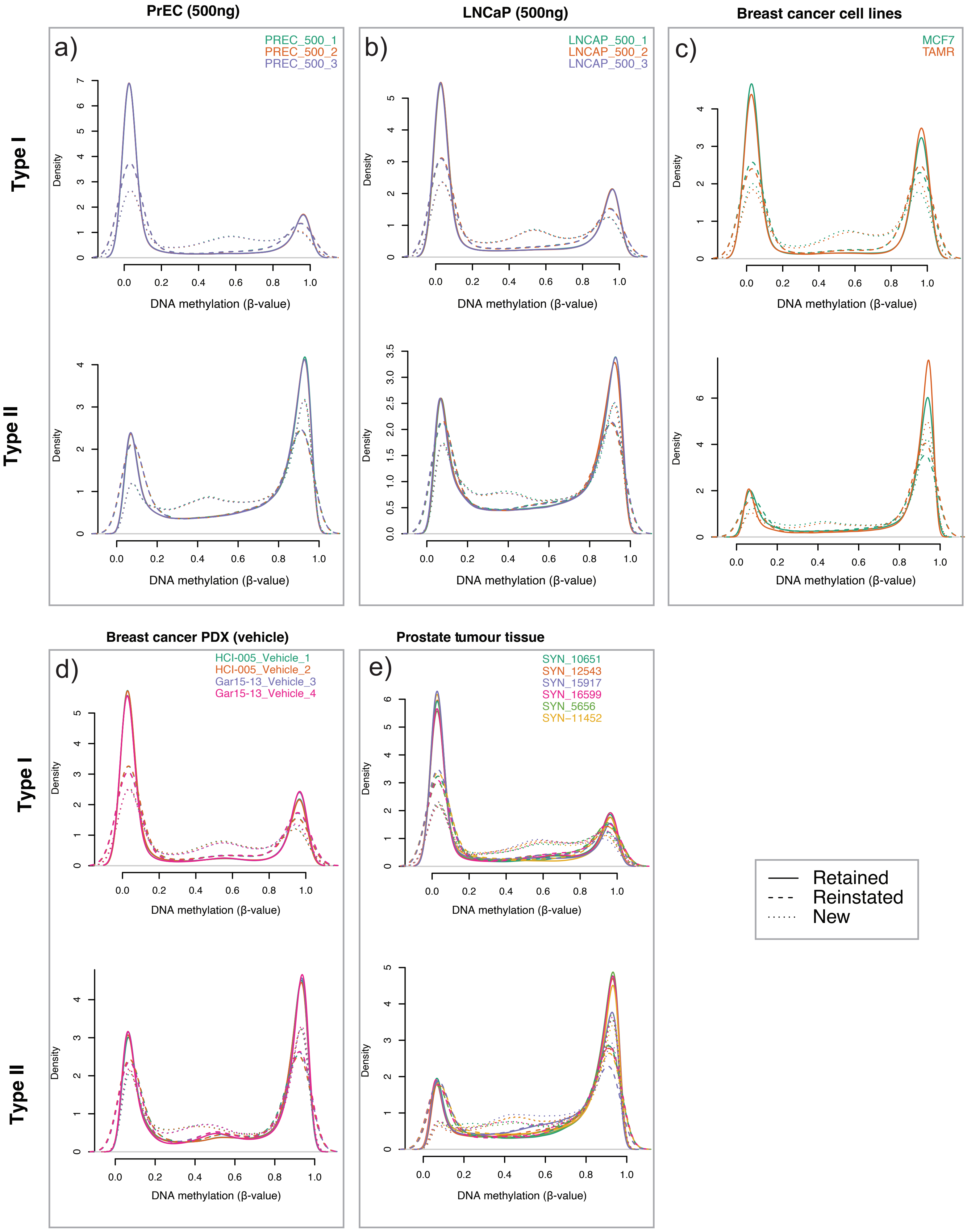

Supplementary Figure 7) Density plot of methylation values for samples profiled on EPICv2 split by both Infinium probe design and probe category (retained, reinstated or new).

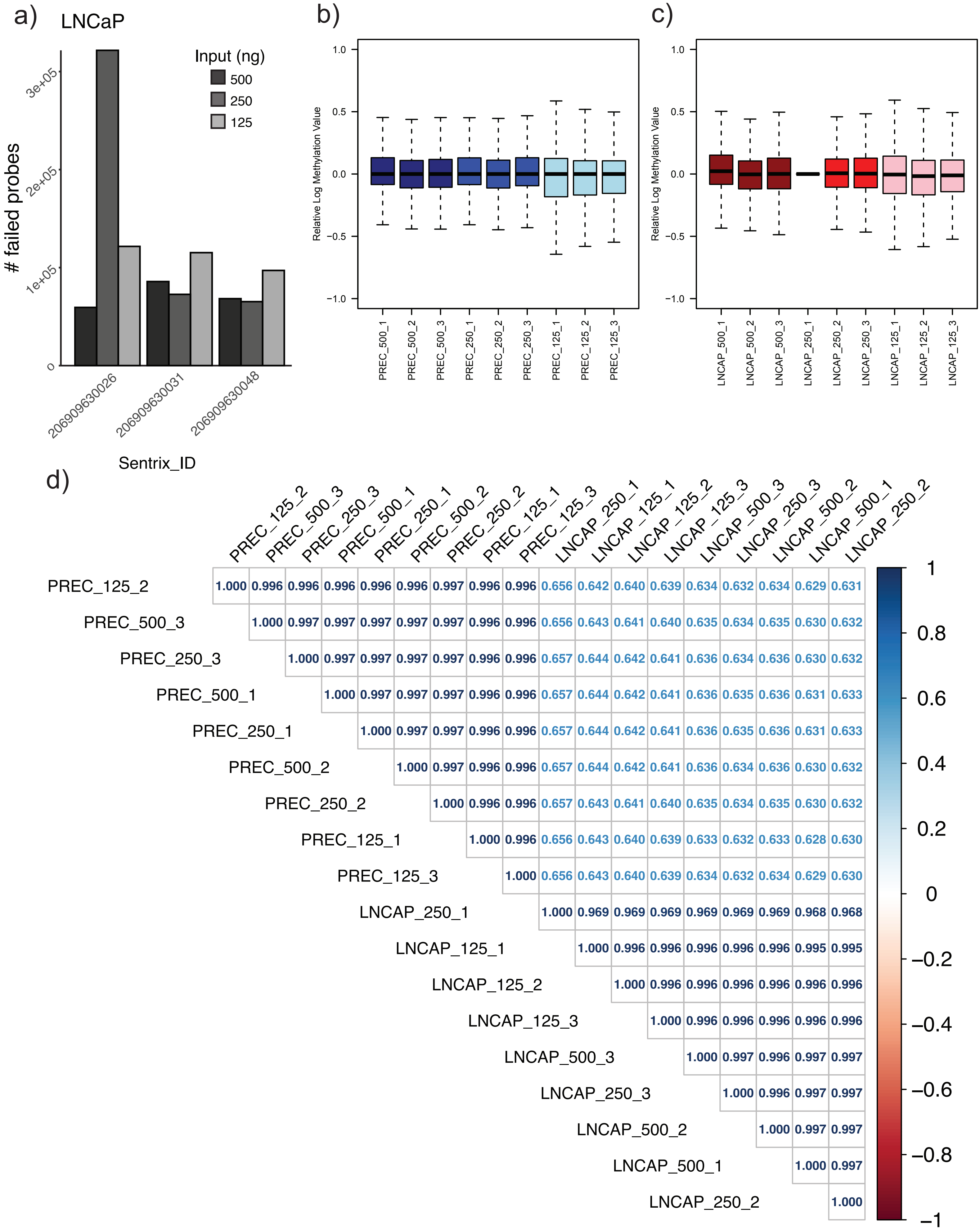

Supplementary Figure 8) Comparison of LNCaP technical replicates with varying DNA input levels showing a) number of probes with detection p-value >0.05, Relative Log Methylation plots of b) PrEC and c) LNCaP and d) correlation matrix of methylation values between technical replicates.

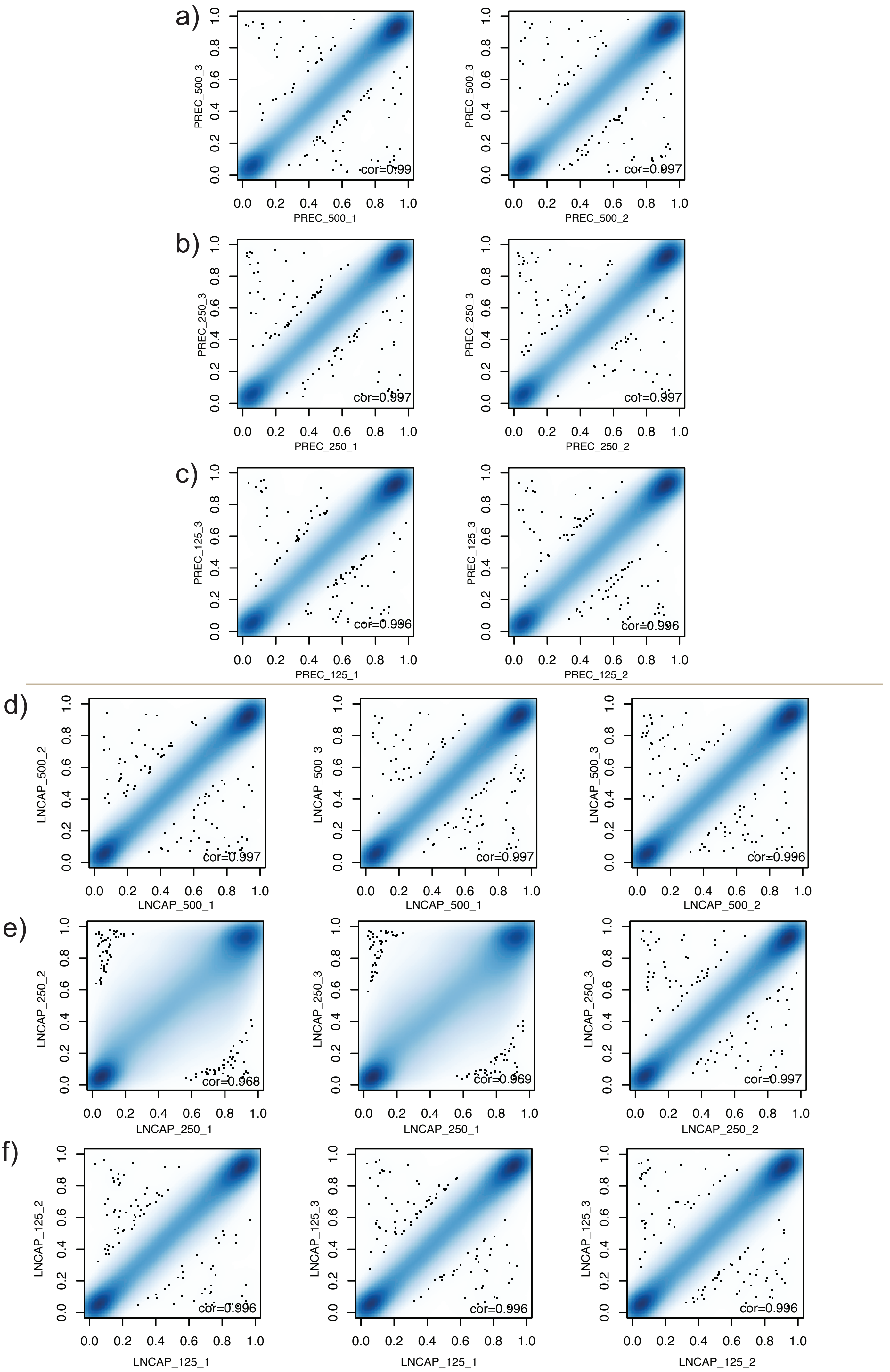

Supplementary Figure 9) Correlation plots of methylation β-value of technical replicates with varying DNA input levels for PrEC a) 500ng, b) 250ng, c) 125ng, and LNCaP d) 500ng, e) 250ng, f) 125ng

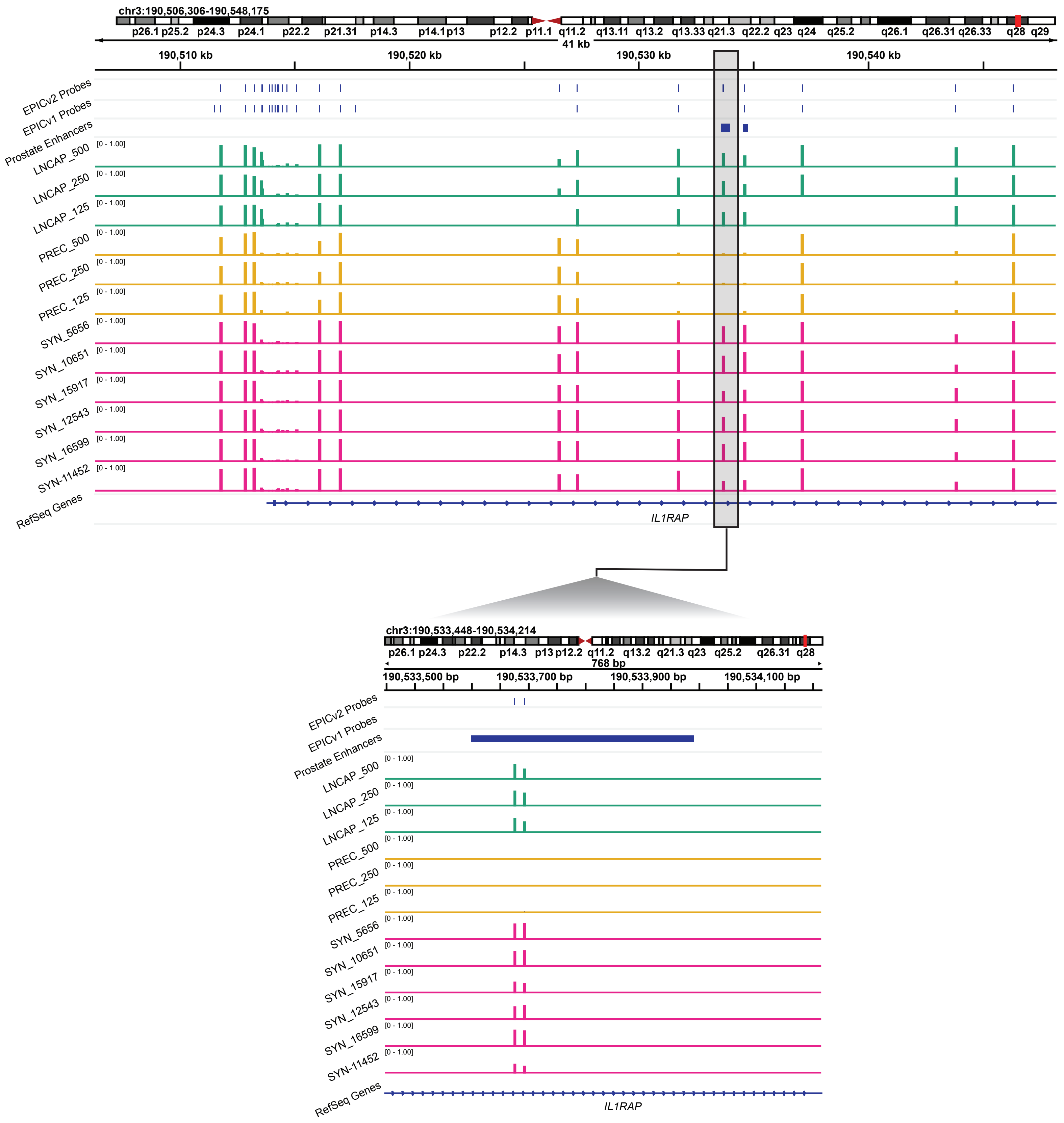

Supplementary Figure 10) Genome browser image showing methylation of prostate cancer tissue and cell line samples (of varying DNA input levels) at new EPICv2 site at FANTOM5 prostate enhancer region.

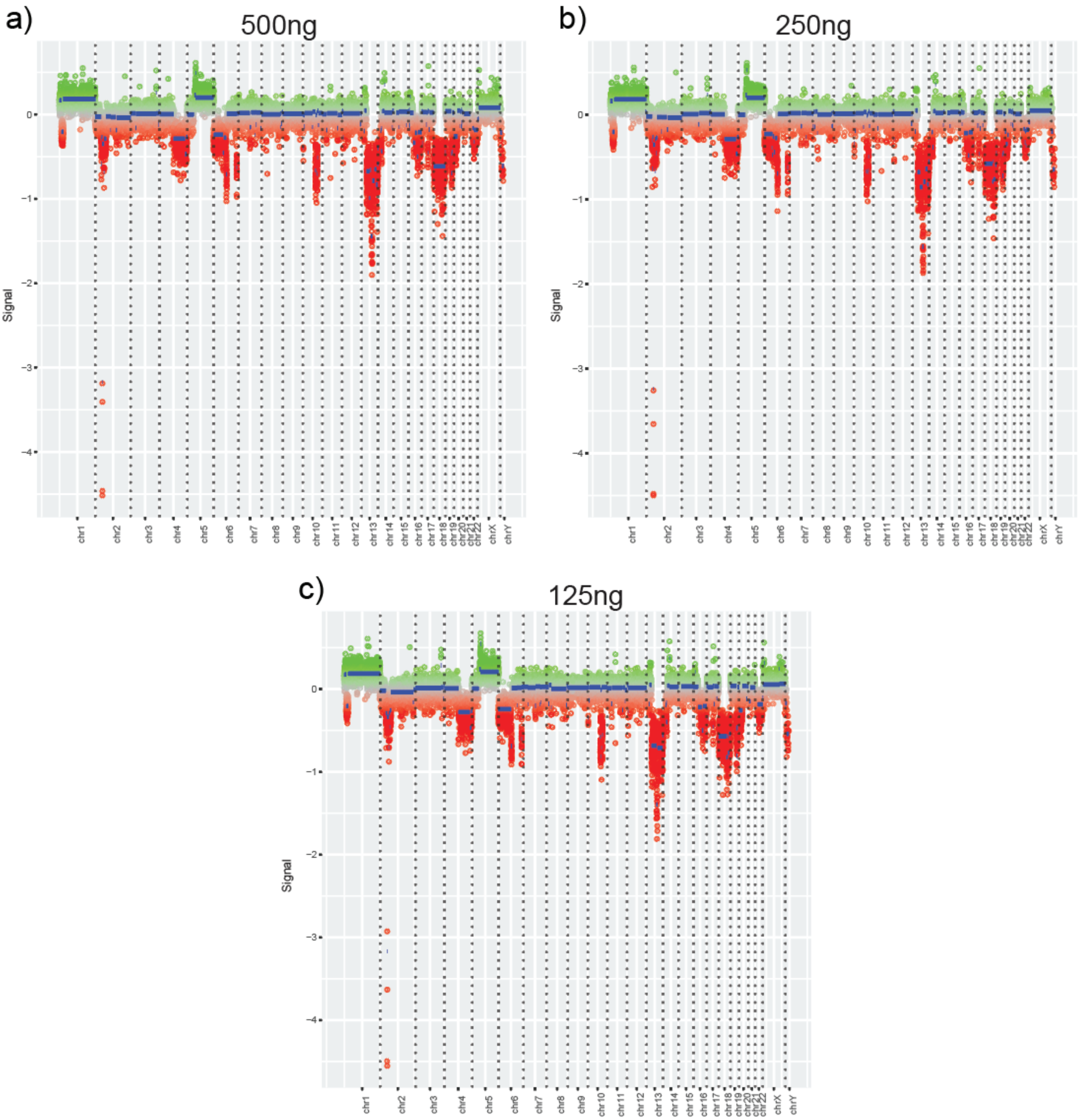

Supplementary Figure 11) Genomic copy number for LNCaP (using PrEC as a reference) at a range of DNA input levels: a) 500ng, b) 250ng and c) 125ng.

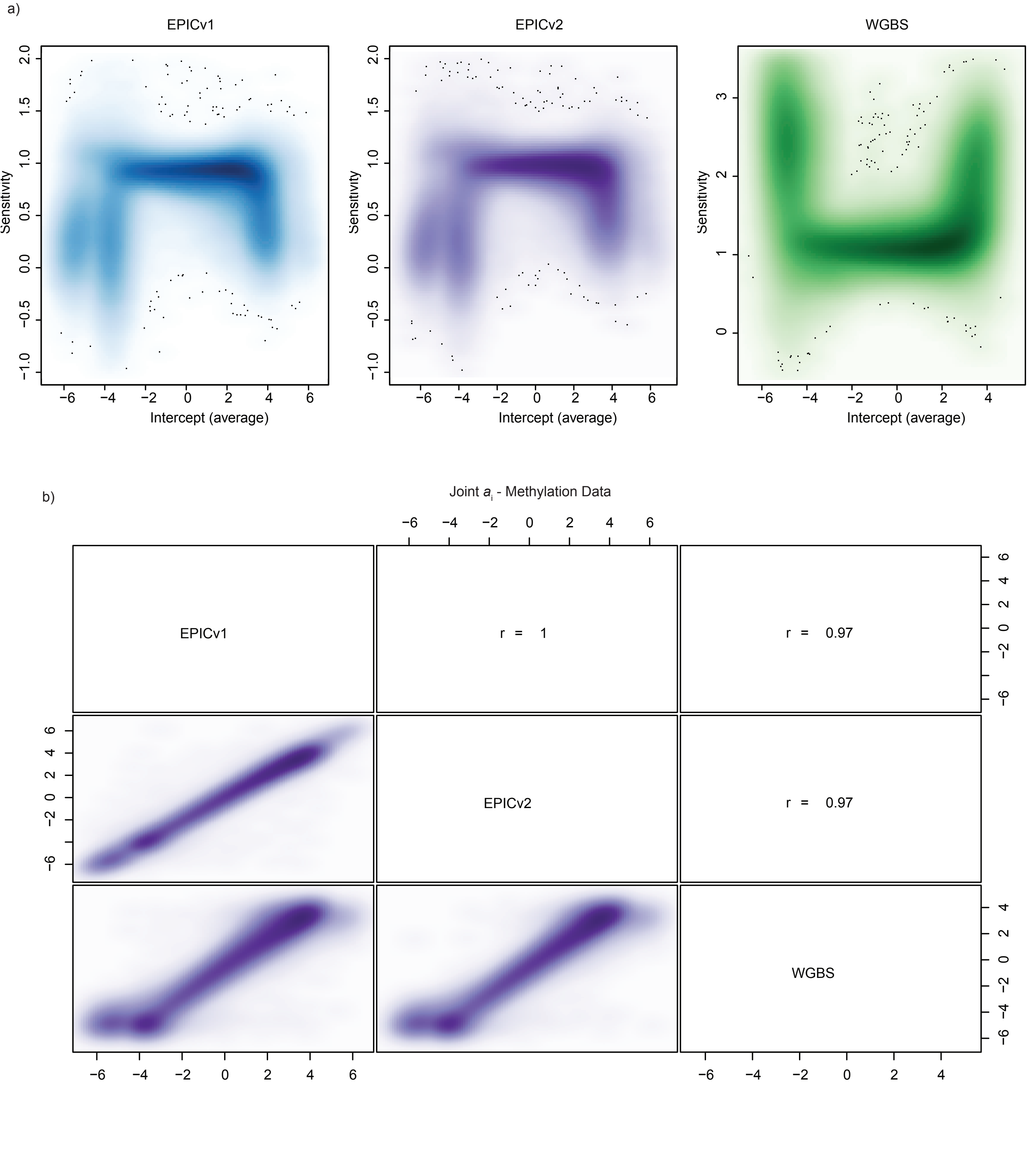

Supplementary Figure 12) a) Sensitivity as a function of row-linear intercept (M-value methylation domain) for the three platforms tested. b) Pairwise joint distributions of platform averages across all CpG sites tested.

a)

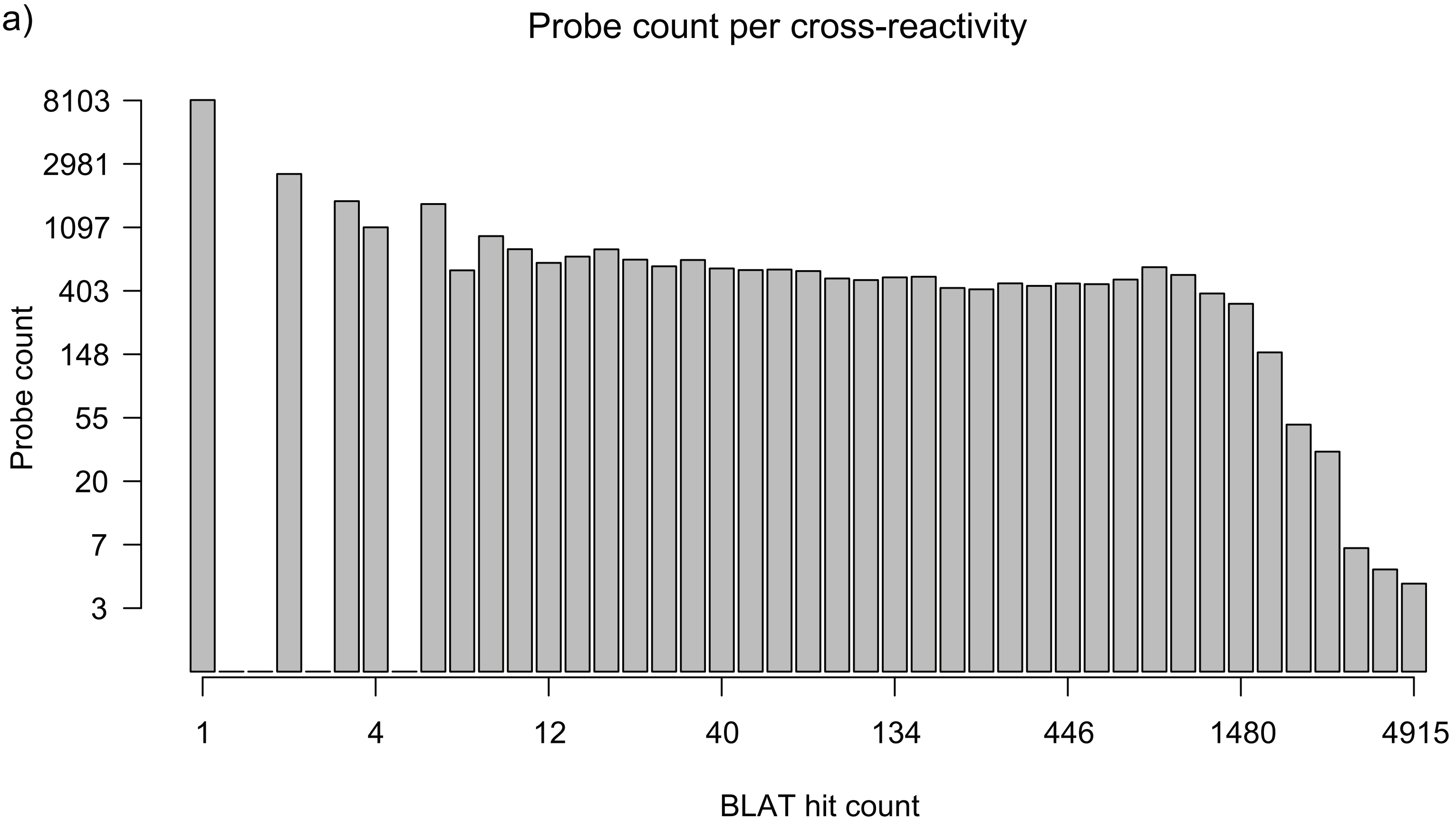

Supplementary Figure 13) Barplot of probe counts by given number of in silico cross-hybridisation events via BLAT. Note log transform for both axes.

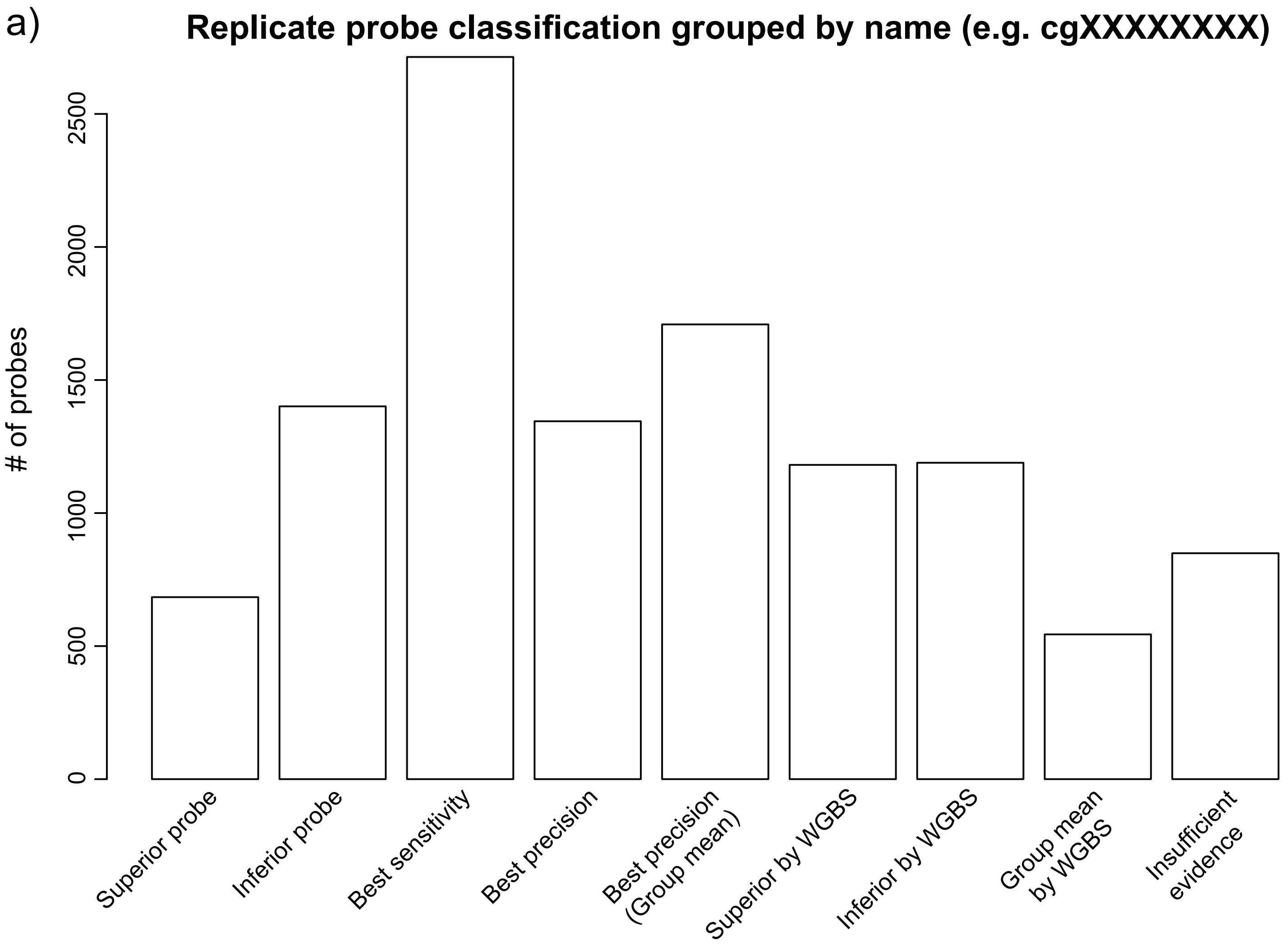

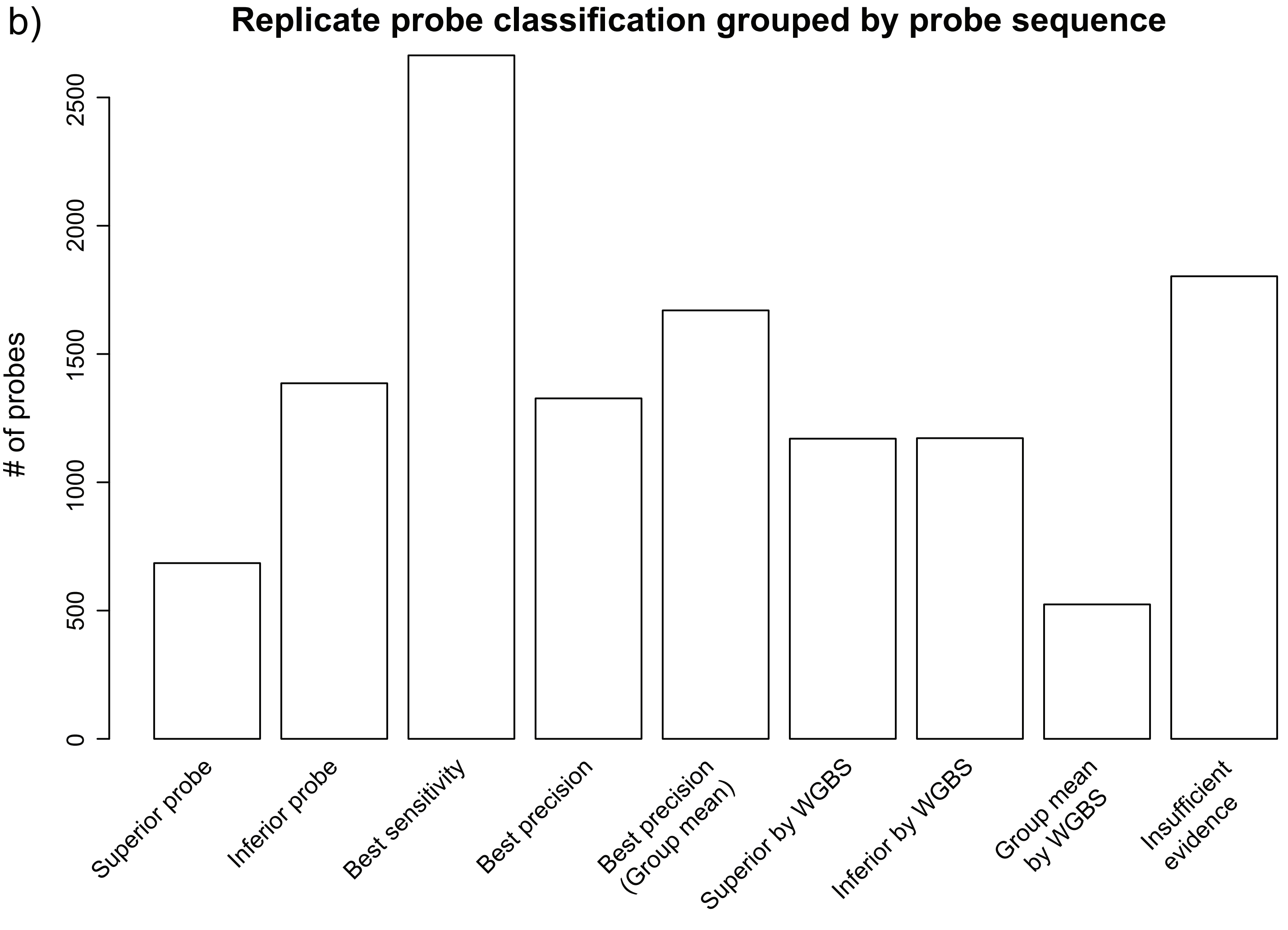

Supplementary Figure 14) Barplot of results from competitive comparison of probe replicates, where sets are grouped by a) IlmnID and b) probe sequence.
