# Supplementary material for: Characterisation and reproducibility of the HumanMethylationEPIC v2.0 BeadChip for DNA methylation profiling
Source: BMC Genomics. 2024 Mar 6;25:251. doi: 10.1186/s12864-024-10027-5 (PMC10916044; doi:10.1186/s12864-024-10027-5)
Supplement: Supplementary file 7 — Additional file 7. Supplementary Note. A pdf containing additional results about the genomic distribution of EPICv2 probes relative to chromosomal location. [file 12864_2024_10027_MOESM7_ESM.pdf]

Supplementary Note: Analysis of EPICv2 probe chromosomal distribution

The EPICv2 probe sites are distributed throughout the genome, targeting all autosomal chromosomes, sex chromosomes and mitochondrial DNA, ranging from 87,022 sites on chromosome 1 to nine sites on chromosome M (**Figure A**, **Additional File 1: Table S11**). Normalizing the number of EPICv2 probe sites per million base pairs on each chromosome, shows that chromosome 17, 19 and 22 are relatively enriched for probes compared to other chromosomes (**Figure B**).

In comparison with the 'retained' sites from EPICv1, the 'new' sites targeted by EPICv2 are relatively enriched for chromosome 9 (5.9 % of 'new' locations versus 3.0 % of 'retained' locations), as well as chromosomes 13, 18, 20, 21, 22, X, Y and M (**Figure C**, **Additional File 1: Table S11**). All nine sites on chromosome M are uniquely targeted by the EPICv2, with the previous nine chromosome M probes targeted by EPICv1 now excluded. The 'reinstated' locations are relatively enriched for chromosomes 1, 2, 6, 7, 8, 10 X and Y, compared to the 'retained' locations (for example, 10.3 % of 'reinstated' locations versus 6.1 % of 'retained' locations are on chromosome 6). This in part compensates for the probe locations excluded between EPICv1 and EPICv2, which were a higher proportion of chromosome 6 and 7 sites (for example 7.0 % of 'excluded' sites were on chromosome 6 versus 6.1 % of 'retained' sites).

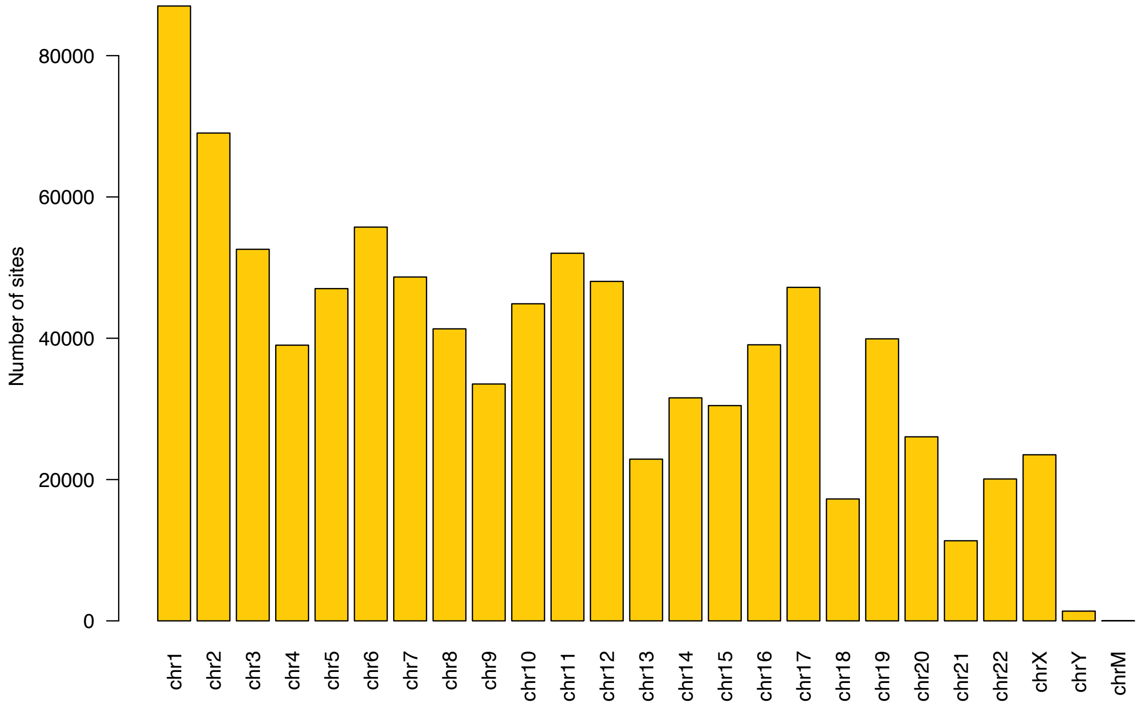

Figure A: Number of sites targeted by EPICv2 probes on each chromosome (hg38)

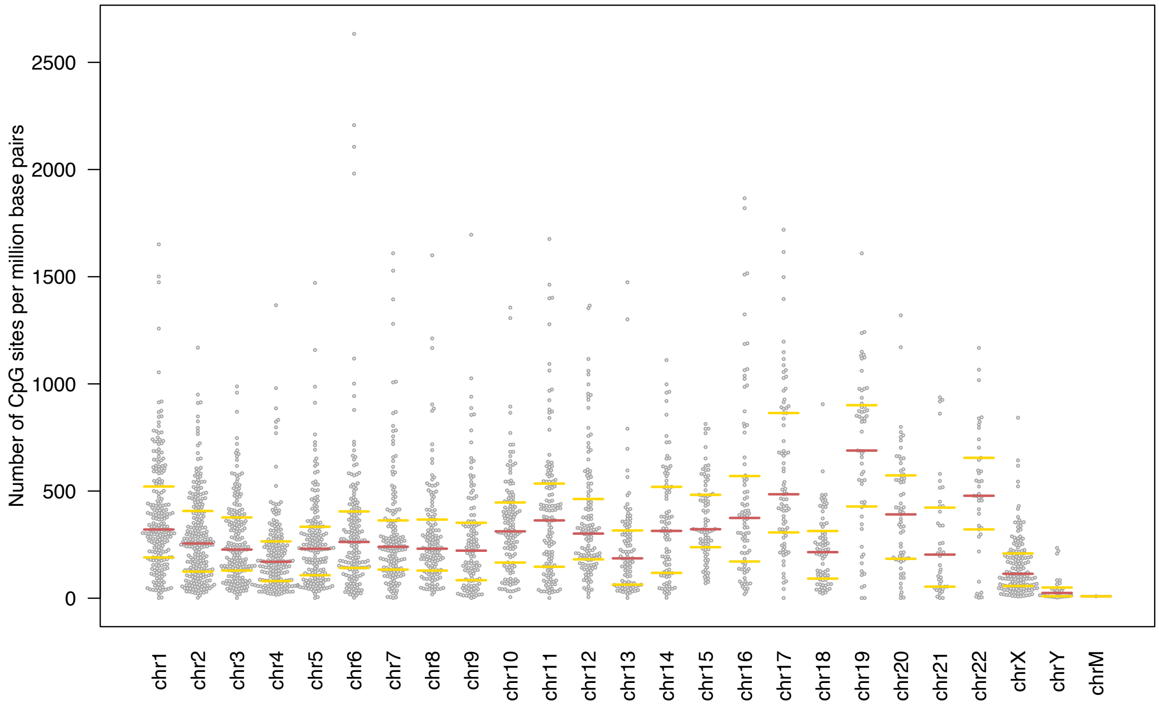

Figure B: Number of sites targeted by EPICv2 probes on each chromosome (hg38)

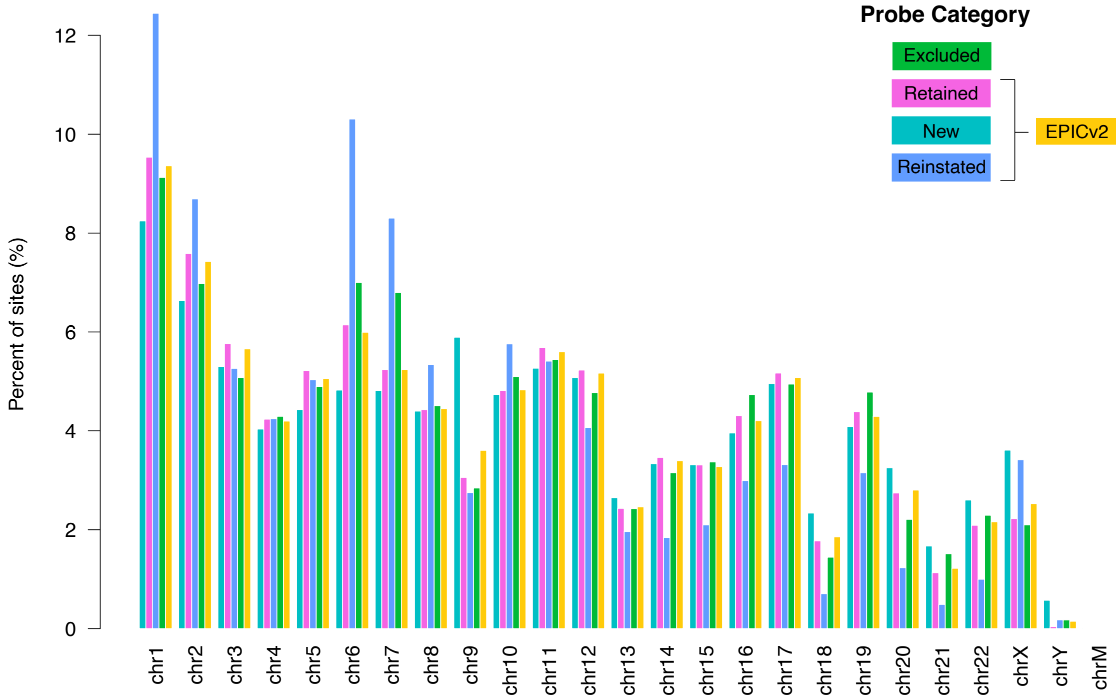

Figure C: Percent of sites (per probe category) targeted by EPICv2 probes on each chromosome (hg38)
